# Metabolic reprogramming is critical to microglial activation in Huntington’s disease

**DOI:** 10.1172/jci.insight.201466

**Published:** 2026-04-02

**Authors:** Abhishek Jauhari, Adam C. Monek, Olena S. Abakumova, Tanisha Singh, Sukhman Singh, Xiaomin Wang, Carley S. Clise, Diane L. Carlisle, Robert M. Friedlander

**Affiliations:** 1 Neuroapoptosis Laboratory, Department of Neurological Surgery, University of Pittsburgh, School of Medicine, Pittsburgh, Pennsylvania, USA.; 2 Helexva Inc., Pittsburgh, Pennsylvania, USA.

**Keywords:** Metabolism, Neuroscience, Glucose metabolism, Neurodegeneration

## Abstract

Huntington’s disease (HD) is a fatal neurodegenerative disease caused by an expanded polyglutamine (CAG) repeat in the N-terminal of the huntingtin protein (*HTT*). Microglial activation and elevated proinflammatory cytokines are observed in HD brains, but the mechanisms regulating neuroinflammation and microglial activation are poorly understood. Metformin-mediated neuroprotection has been demonstrated in experimental models of neurodegeneration, including HD. We found that metformin inhibits mitochondrial DNA (mtDNA) release and subsequent neuroinflammation in the cortex and striatum of a mouse model of HD. Moreover, elevated proinflammatory cytokines and microglial activation are inhibited by metformin in HD transgenic mouse brains. Metformin reduced pathological microglial clusters and shifted toward a quiescent, homeostatic phenotype. Metformin improved aberrant immunometabolism in HD mouse brains and primary microglia. Mechanistically, we found that metformin regulates mitochondrial fission, reprograms deregulated metabolism in HD microglia, and controls microglial activation and inflammation in HD transgenic mice.

## Abstract 2

N/A

## Graphical abstract

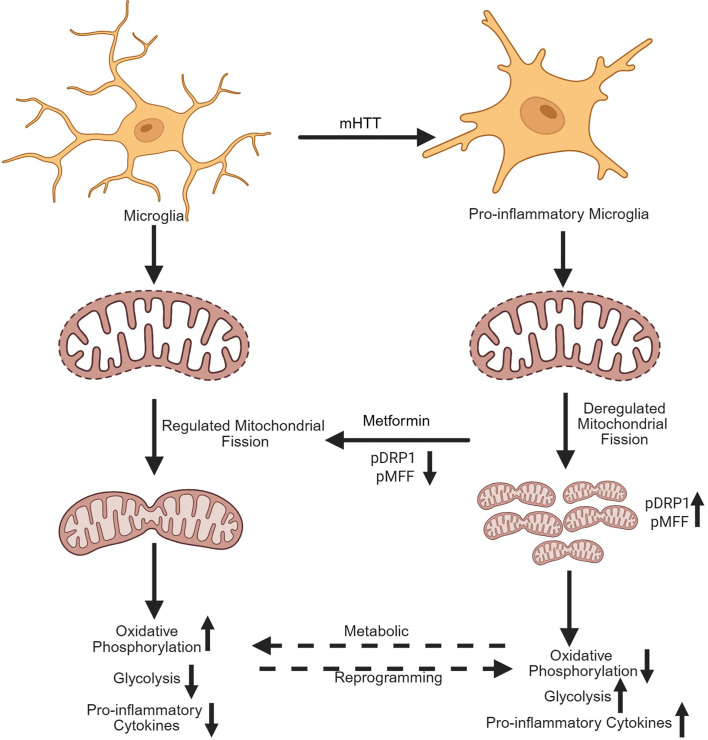

## Introduction

Huntington’s disease (HD) is an autosomal-dominantly inherited neurodegenerative disorder caused by expanded CAG trinucleotide repeats (>36) in exon 1 of the *HTT* gene, encoding the huntingtin protein (1, 2). Mutant huntingtin (mHTT) accumulation in microglia has been demonstrated in mice and human HD samples (3–5). Microglia, the resident immune cells of the central nervous system, play a crucial role in maintaining brain homeostasis. In response to neuroinflammatory signals, microglia transition from a quiescent state to an activated state, exhibiting diverse immune functions such as phagocytosis, cytokine release, and reactive oxygen species (ROS) production (6). Microglial activation is characterized by complex transcriptional, epigenomic, and functional changes triggered by various stimuli, including pathogen- and microbe-associated molecular patterns (such as LPS), damage-associated molecular patterns, interferons (IFNs), and interleukins (ILs) (7–11). While microglial activation plays a central role in neuroinflammation and synaptic degeneration, it is tightly regulated in both healthy and diseased brains. Activated microglia undergo rapid morphological and molecular changes in response to pathological insults (12).

Microglial activation is often described along a spectrum ranging from proinflammatory to antiinflammatory or reparative states, as well as quiescent or homeostatic phenotypes. Proinflammatory microglial states release cytokines such as IL-1β, IL-6, and TNF-α, whereas antiinflammatory or reparative states express factors including TGFB, CD206, and Arg1 (13). Striking a balance between these states is essential for modulating neuroinflammation. Proinflammatory microglial activation is commonly observed in neurodegenerative diseases, including Alzheimer’s, Parkinson’s, and HD (14–16). Caspase-1 inhibition and associated reduction of mature IL1B generation have been demonstrated to be neuroprotective. Furthermore, the reduction of microglial activation is also associated with neuroprotection in vivo (17–21). Activated microglia also exhibit enhanced phagocytic activity, engulfing degenerated synapses and contributing to synaptic plasticity (22–24). However, their effects can be either neurotoxic or neuroprotective, depending on the context (3, 25).

Microglial metabolism is highly dynamic, shifting between glycolysis and oxidative phosphorylation (OXPHOS) in response to environmental cues. This metabolic reprogramming profoundly influences microglial activation and immune function. For instance, a shift toward glycolysis promotes the production of proinflammatory cytokines, prostaglandins, and chemokines, enhancing microglial pathogenic activity (26–29). Conversely, proper mitochondrial function and normal reliance on OXPHOS-mediated energy production suppress glycolytic by-product production and inhibit microglial activation (27). The shift to glycolysis from OXPHOS in HD is likely mediated by the early mitochondrial dysfunction detected in R6/2 mice, directly related to mutant HTT binding to the mitochondrial protein transporter TIM23, resulting in mitochondrial proteome dyshomeostasis (30–32). An additional stress on mitochondria in HD results from a melatonin synthetic deficit, which can be mitigated by melatonin supplementation (33).

Metformin, a widely used antidiabetic drug, has antiinflammatory and antineoplastic properties (34–37). Recent studies highlight its neuroprotective potential in neurodegenerative diseases, including HD. Metformin appears to mitigate neurodegeneration by regulating energy metabolism, reducing oxidative stress, modulating inflammatory responses, and preventing pathological protein aggregation. In HD transgenic mice, metformin improves motor function and survival, likely through its effects on energy homeostasis and immunometabolism (34, 38–42). The neuroprotective mechanisms of metformin are mediated in part by its activation of adenosine monophosphate–activated protein kinase (AMPK), a key regulator of cellular energy balance and longevity. AMPK activation is associated with neuroprotection in HD models, both in vitro and in vivo (43, 44). Notably, metformin reduces mHTT nuclear aggregation in the striatum, partially restores neurotrophic factor expression, and decreases glial activation in the zQ175 mouse model of HD (45). Here, we demonstrate that metformin modulates immunometabolism and microglial activation in HD transgenic mice.

## Results

### Metformin inhibits mtDNA release and subsequent inflammation in R6/2 mice.

**Figure 1:**
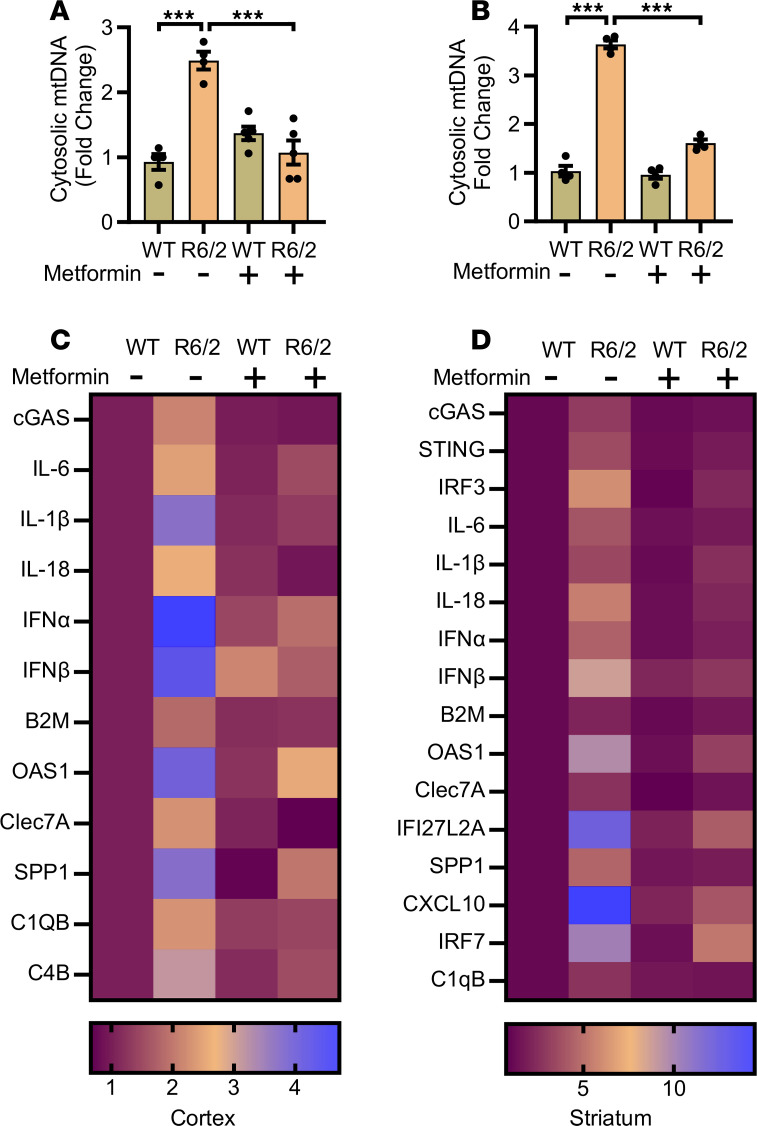
Metformin regulates mtDNA release and subsequent inflammation in HD. (**A**) Quantification of cytosolic mtDNA in WT and R6/2 cortex treated with or without metformin; *n* = 4 for vehicle and *n* = 5 for metformin group. (**B**) Quantification of cytosolic mtDNA in WT and R6/2 striatum treated with or without metformin; *n* = 4. (**C**) Heatmap showing qPCR-based mRNA expression of inflammatory, microglial activation, and synaptic genes in the cortex of WT and R6/2 mice, demonstrating that genes significantly upregulated in R6/2 mice were significantly downregulated following metformin treatment; *n* = 3–5. (**D**) Heatmap showing qPCR-based mRNA expression of inflammatory, microglial activation, and synaptic genes in the striatum of WT and R6/2 mice, demonstrating that genes significantly upregulated in R6/2 mice were significantly downregulated following metformin treatment. WT and R6/2 mice were treated with metformin 200 mg/kg body weight intraperitoneally. Data are represented as mean ± SEM. Individual data points in the graphs represent an independent biological sample; *n* = 3–5. Data were analyzed by 2-way ANOVA followed by Tukey’s test. ***P < 0.001.

We previously demonstrated that mitochondrial DNA (mtDNA) release into the cytosol activates the cGAS/STING pathway, leading to an inflammatory response in HD (R6/2) and accelerated-aging (*AANAT*-KO) mouse brains (46). Inhibition of mtDNA release by melatonin supplementation reduces proinflammatory cytokine levels in the brains of HD mice (46). Metformin is known to attenuate neuroinflammation via TREM2/SYK and TRPV1/NLRP3 pathways, alleviating central sensitization in migraine and depressive-like behaviors in allergic rhinitis models (47, 48). Therefore, we evaluated whether metformin inhibited mtDNA release from mitochondria to the cytosol and the subsequent inflammatory responses in the cortex and striatum of R6/2 mice, which express mutant human huntingtin exon 1 with expanded CAG repeats and show HD-like symptoms and neurodegeneration (49). Five- to six-week-old wild-type (WT) and R6/2 mice (presymptomatic/early symptomatic) were treated with metformin for 4 weeks. Ten-week-old R6/2 mice are in their mid-symptomatic stage. As previously reported, a significant increase in mtDNA release was observed in the cortex and striatum of R6/2 mice, and this release was inhibited by metformin (Figure 1, A and B, and Supplemental Figure 1, A and B; supplemental material available online with this article; https://doi.org/10.1172/jci.insight.201466DS1). We then evaluated the impact of metformin on mtDNA-mediated signaling, microglial activation, synaptic degeneration, and proinflammatory cytokine gene expression. Increased expression of genes related to mtDNA signaling (*cGAS* in cortex and *cGAS*, *STING*, and *IRF3* in striatum), proinflammatory cytokines (*IL6*, *IL1B*, *IL18*, *IFNA*, and *IFNB* in both cortex and striatum), and microglial activation markers (*B2M*, *OAS1*, *CLEC7A*, and *SPP1* in cortex and *B2M*, *OAS1*, *CLEC7A*, *IFI27L2A*, *SPP1*, and *CXCL10* in striatum) was observed in 10-week-old R6/2 mice. Metformin suppressed these changes in both the cortex (Figure 1C and Supplemental Table 1) and striatum (Figure 1D and Supplemental Table 2).

### Metformin inhibits proinflammatory microglial activation in R6/2 mice.

**Figure 2:**
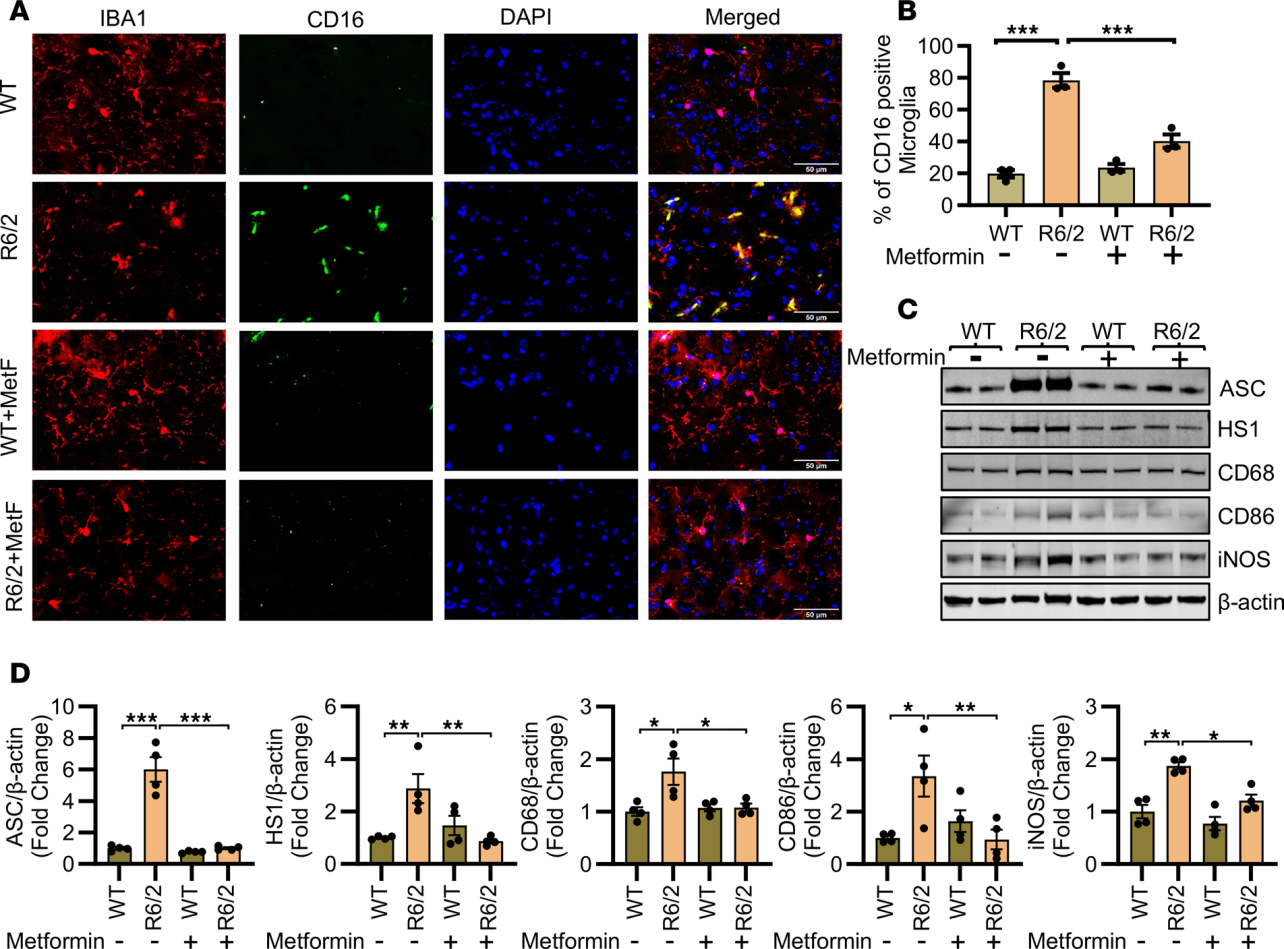
Metformin inhibits proinflammatory microglial activation in HD. (**A**) Representative immunofluorescence images stained with IBA1 (microglia marker), CD16 (proinflammatory microglia marker), and DAPI (nuclear marker) in WT and R6/2 striatum treated with metformin (200 mg/kg body weight) or vehicle. Scale bars: 50 μm. (**B**) Quantification of immunofluorescence in percentage of microglial cells showing CD16 signals per view fields in WT and R6/2 striatum treated with metformin (200 mg/kg body weight) or vehicle; *n* = 3. Twelve images per animal. (**C**) Representative immunoblot images of ASC, HS1, CD68, CD86, iNOS, and β-actin in WT and R6/2 striatum treated with metformin (200 mg/kg body weight) or vehicle. (**D**) Normalized quantification of immunoblots of ASC, HS1, CD68, CD86, and iNOS shown in **C**. Data are represented as mean ± SEM. Individual data points in the graphs represent an independent biological sample. Data were analyzed by 2-way ANOVA followed by Tukey’s test. *P < 0.05; **P < 0.01; ***P < 0.001.

Gene expression analysis revealed that metformin treatment tended to normalize mRNA levels associated with proinflammatory cytokines and synaptic markers in the cortex and striatum of symptomatic R6/2 mice (Figure 1, C and D). Given the elevated levels of proinflammatory cytokine mRNA and the known microglial activation in R6/2 mouse brain (50, 51), we evaluated the impact of metformin treatment on proinflammatory microglia in the R6/2 striatum. Immunofluorescence analysis using IBA1 (a microglial marker) and CD16 (a marker for phagocytic and inflammatory microglia) (52) demonstrated increased colocalization of CD16 and IBA1 in R6/2 striatum in comparison with WT mice (Figure 2, A and B). These findings suggest increased phagocytic and proinflammatory microglial activation in HD (3, 50, 53), and metformin treatment reduced this activation to near WT levels (Figure 2, A and B). Further, immunoblot analysis of proinflammatory microglial markers, including ASC/TMS1, HS1, CD68, CD86, and iNOS (52), revealed increased protein levels in the R6/2 striatum. Metformin normalized these levels to those observed in WT mice (Figure 2, C and D). Immunostaining and immunoblot analysis together demonstrated increased proinflammatory microglial activation in R6/2 brains, and this response was attenuated by metformin treatment.

**Figure 3:**
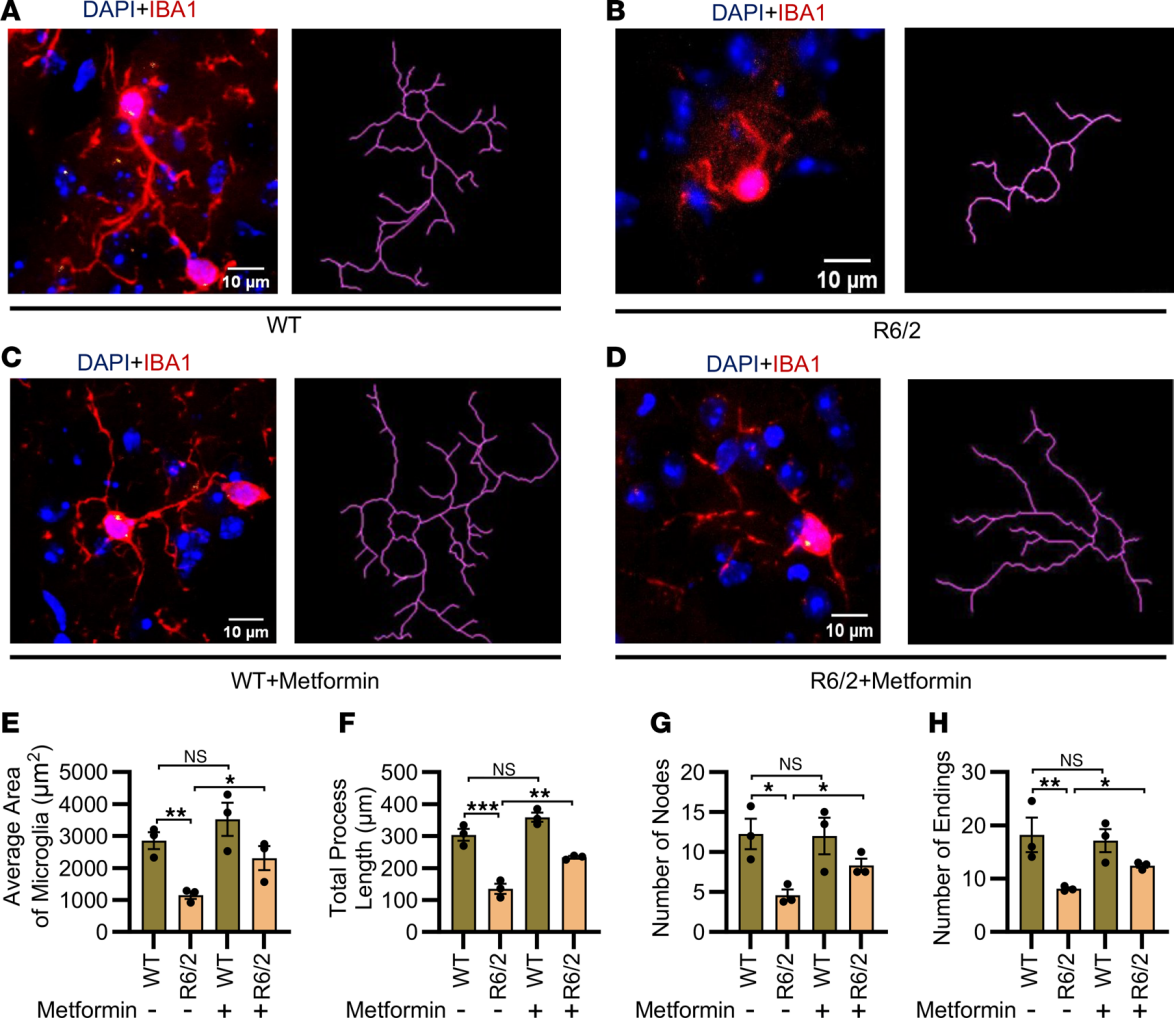
Metformin regulates microglial ramification in HD. (**A**–**D**) Representative images of microglial morphology traced with NeuronJ of ImageJ from the striatum of WT brain (**A**), striatum of R6/2 brain (**B**), striatum of WT brain treated with metformin (**C**), and striatum of R6/2 brain treated with metformin (**D**). Note that microglial cells from untreated R6/2 mice display shorter processes and diminished ramification of processes (**B**), whereas microglia from metformin-treated R6/2 mice show longer and highly ramified processes (**D**). Scale bars: 10 μm. (**E**) Quantification of average area occupied by individual microglia (in μm^2^) in the striatum of WT and R6/2 mice treated with metformin or vehicle. (**F**) Quantification of total process length by individual microglia (in μm) in striatum of WT and R6/2 mice treated with metformin or vehicle; *n* = 3. (**G**) Quantification of number of nodes in individual microglia in striatum of WT and R6/2 mice treated with metformin or vehicle. (**H**) Quantification of number of endings in individual microglia in the striatum of WT and R6/2 mice treated with metformin or vehicle (10–14 microglial cells per animal, *n* = 3 animals per group). Data are represented as mean ± SEM. Individual data points in the graphs represent an independent biological sample. Data were analyzed by 2-way ANOVA followed by Tukey’s test. *P < 0.05; **P < 0.01; ***P < 0.001.

To further investigate microglial activation and the effect of metformin, we analyzed microglial morphology in the R6/2 striatum. Using the NeuronJ plug-in in ImageJ (NIH) and IBA1-immunostained brain sections, we traced individual microglial processes in the striatum of WT and R6/2 mice, with and without metformin treatment. Morphologically, most IBA1^+^ microglia in the R6/2 striatum exhibited an activated phenotype, characteristic of proinflammatory phagocytic microglia, with reduced process ramification and number compared with age-matched WT controls (Figure 3, A and B). In contrast, microglia in the metformin-treated R6/2 striatum displayed a quiescent, homeostatic phenotype, characterized by highly ramified processes that occupied a larger area of brain tissue (Figure 3C), with no significant changes observed in WT mice following metformin treatment (Figure 3D).

Quantitative analysis confirmed the protective antiinflammatory effects of metformin treatment in the R6/2 striatum, as evidenced by increased area occupied by individual microglia (Figure 3E), enhanced total process length (Figure 3F), and a higher number of nodes and endings (Figure 3, G and H). These results provide evidence that metformin mitigates the phenotypic and functional characteristics of proinflammatory microglia.

### Metformin reprograms impaired metabolism in R6/2 mouse brain.

**Figure 4:**
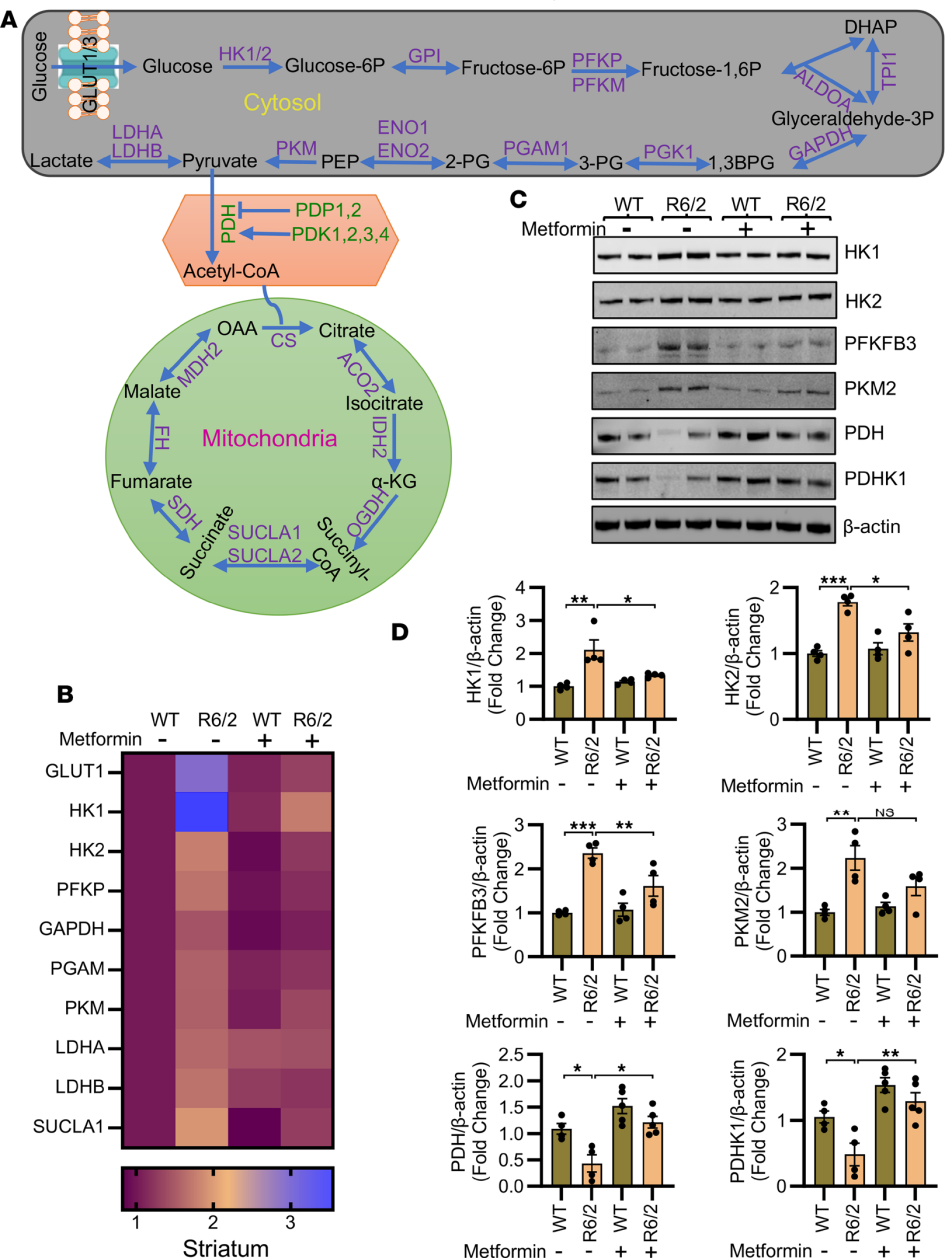
Metformin reprograms impaired metabolism in the R6/2 mouse brain. (**A**) Pictorial representation of glucose metabolism. (**B**) Heatmap showing qPCR-based mRNA expression of glucose glycolytic metabolic enzymes in the striatum of WT and R6/2 mice, demonstrating that genes significantly altered in R6/2 mice were significantly modulated following metformin treatment; *n* = 4. (**C** and **D**) Representative immunoblot images of hexokinase 1 (HK1), hexokinase 2 (HK2), PFKFB3, PKM2, pyruvate dehydrogenase (PDH), PDHK1, and β-actin (**C**) and their normalized expression levels (**D**) in WT and R6/2 striatum treated with vehicle or metformin (200 mg/kg body weight for 4 weeks); *n* = 4 for HK1, HK2, PFKFB3, and PKM2 and *n* = 5 for metformin group for PDH and PDHK1. Data are represented as mean ± SEM. Individual data points in the graphs represent an independent biological sample. Data were analyzed by 2-way ANOVA followed by Tukey’s test. *P < 0.05; **P < 0.01; ***P < 0.001.

Microglia are highly responsive to their microenvironment and dynamically modulate metabolic pathways and immune properties in response to various stimuli (54). Inflammatory signals reprogram microglial metabolism, a key feature being increased glycolytic flux. Given this, we determined whether metformin influenced metabolic pathways in R6/2 brains. Glycolysis converts glucose to pyruvate through a series of steps, with pyruvate subsequently metabolized to acetyl-coenzyme A (acetyl-CoA), which enters the mitochondria to activate OXPHOS (Figure 4A) (55). To examine metabolic deregulation in R6/2 brains and the modulatory effects of metformin, we analyzed the gene expression profiles of glycolytic and OXPHOS enzymes in striatum (Figure 4B and Supplemental Table 3). This analysis revealed that the expression of the glucose transporter GLUT1 and glycolytic enzymes — hexokinases (*HK1* and *HK2*), phosphofructokinase (*PFKP*), glyceraldehyde-3-phosphate dehydrogenase (*GAPDH*), phosphoglycerate mutase (*PGAM*), pyruvate kinase M (*PKM*), and lactate dehydrogenase A and B (*LDHA* and *LDHB*) — was elevated in the R6/2 striatum, and this increase was significantly inhibited by metformin (Figure 4B and Supplemental Table 3). Notably, we did not find any significant alterations in the enzymes’ mRNAs involved in the conversion of pyruvate to acetyl-CoA (Supplemental Table 3). In the OXPHOS pathway, aconitase 2 (*ACO2*) was reduced, whereas succinate-CoA ligase α (*SUCLA1*) and components of succinate dehydrogenase complex (*SDHC* and *SDHD*) were increased in R6/2 striatum (Figure 4B and Supplemental Table 3), and metformin only mitigated the level of *SUCLA1* (Figure 4B and Supplemental Table 3). These results suggest deregulation of the glycolysis component in the R6/2 striatum. We therefore evaluated whether the changes of key glycolytic enzymes (hexokinases, phosphofructokinase, pyruvate kinase, pyruvate dehydrogenase, etc.) can also be demonstrated at the protein level. We found higher levels of glycolytic enzymes in R6/2 mouse striatum as compared with WT, which were normalized by metformin as demonstrated at the mRNA level (Figure 4, C and D). We analyzed levels of 2 isoforms of hexokinase (HK1 and HK2), which convert glucose to glucose-6-phosphate as the first step of glycolysis (56), and found a significant increase in HK1 and HK2 in R6/2 mouse brain, which was reduced by metformin (Figure 4, C and D).

We further investigated the level of PFKFB3 (6-phosphofructo-2-kinase/fructose-2,6-biphosphatase 3), an activator of the rate-limiting glycolytic enzyme phosphofructokinase (PFKP) (56). Consistent with the findings for hexokinase, the PFKFB3 level was elevated in the R6/2 mouse striatum and was normalized by metformin (Figure 4, C and D). PKM2, which catalyzes the conversion of phosphoenolpyruvate to pyruvate (56), was also increased in the R6/2 mouse striatum; however, it was not reduced by metformin (*P* = 0.09) (Figure 4, C and D). Interestingly, we observed a reduction in pyruvate dehydrogenase (PDH) levels in the R6/2 mouse striatum, which was ameliorated by metformin (Figure 4, C and D). The decreased PDH levels suggest impaired conversion of pyruvate to acetyl-CoA, a crucial step for initiating OXPHOS. Elevated hexokinase levels coupled with decreased PDH suggest an increased reliance on glycolysis with reduced OXPHOS in the R6/2 mouse striatum. Additionally, we found that pyruvate dehydrogenase kinase 1 (PDHK1), which regulates PDH activity, was also decreased in R6/2 mouse striatum and was normalized by metformin (Figure 4, C and D). Collectively, these results indicate that metformin reprograms the impaired metabolic pathways in the R6/2 mouse striatum, which could be linked to the microglial activation and inflammation observed in HD pathology.

### Metabolic impairment in HD human brain.

**Figure 5:**
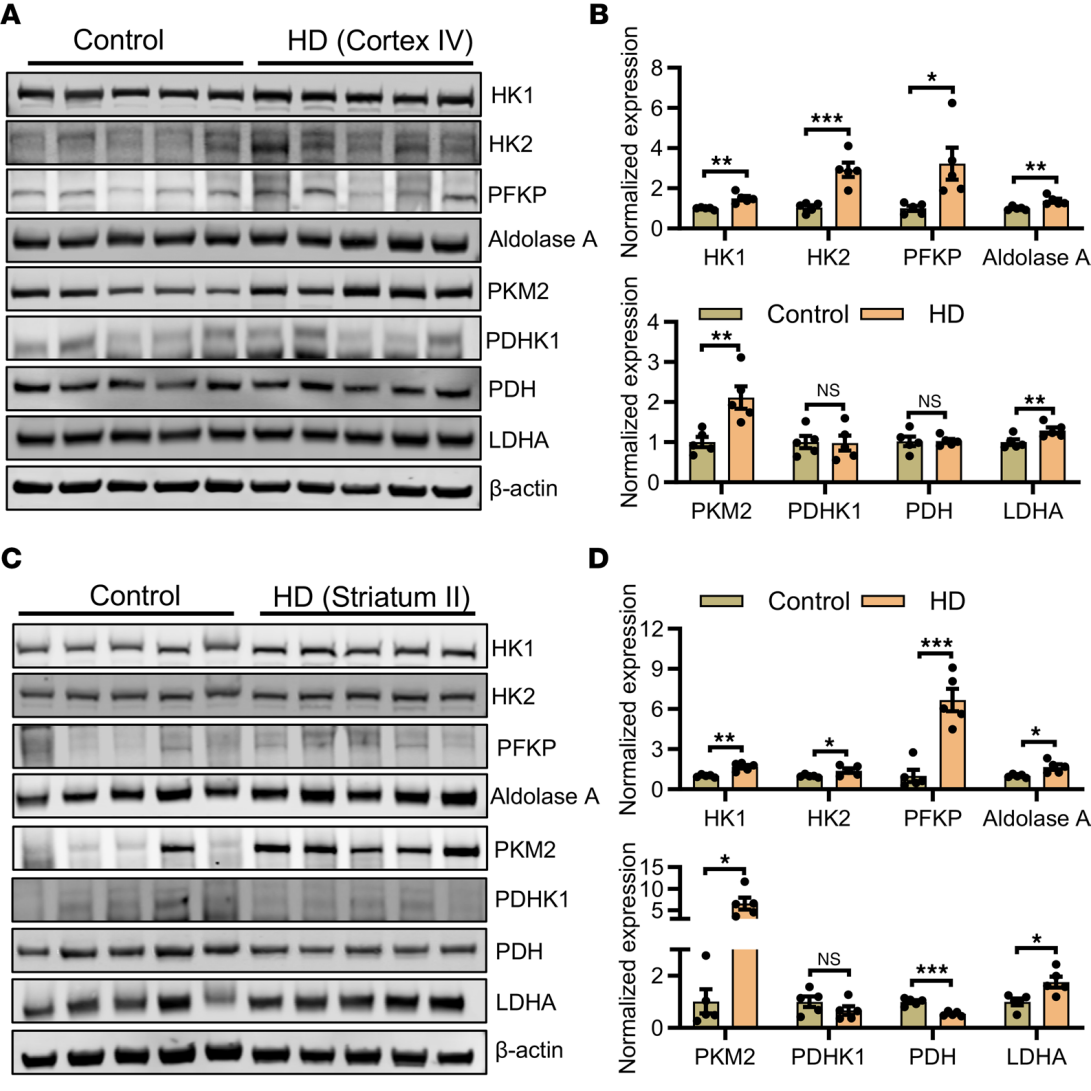
Impaired metabolism in human HD. (**A** and **B**) Immunoblot images of HK1, HK2, PFKP, aldolase A, PKM2, PDHK1, PDH, LDHA, and β-actin (**A**) and their normalized expression levels (**B**) in human postmortem brain cortex of control and HD grade IV; *n* = 5. (**C** and **D**) Immunoblot images of HK1, HK2, PFKP, aldolase A, PKM2, PDHK1, PDH, LDHA, and β-actin (**C**) and their normalized expression levels (**D**) in human postmortem brain striatum of control and HD grade II; *n* = 5. Data are represented as mean ± SEM. Individual data points in the graphs represent an independent biological repeat and were analyzed by 2-tailed Student’s *t* test. *P < 0.05; **P < 0.01; ***P < 0.001.

We observed deregulated glucose metabolism gene expression in the R6/2 striatum, which was restored by metformin (Figure 4). To assess the relevance of these findings to human HD, we examined postmortem human brain samples from HD patients, including grade IV cortex and grade II striatum, alongside age-matched non-neurological controls. Our analysis revealed upregulation of glycolytic enzymes, including HK1, HK2, PFKP, aldolase A, PKM2, and LDHA, in both HD cortex and striatum compared with their age-matched controls (Figure 5, A and B). Similarly, HK1, HK2, PFKP, aldolase A, PKM2, and LDHA were significantly upregulated in the HD striatum compared with age-matched controls (Figure 5, C and D). Notably, PDH was significantly reduced in the HD striatum (Figure 5, C and D). The elevated levels of glycolytic enzymes suggest increased glycolytic flux in HD, while the reduced PDH indicates impaired conversion of pyruvate to acetyl-CoA, pointing to a disruption in OXPHOS. These results highlight metabolic impairment in the HD cortex and striatum, potentially linked to proinflammatory microglial activation.

### Metformin modulates mitochondrial fission to enhance OXPHOS and suppress microglial activation.

**Figure 6:**
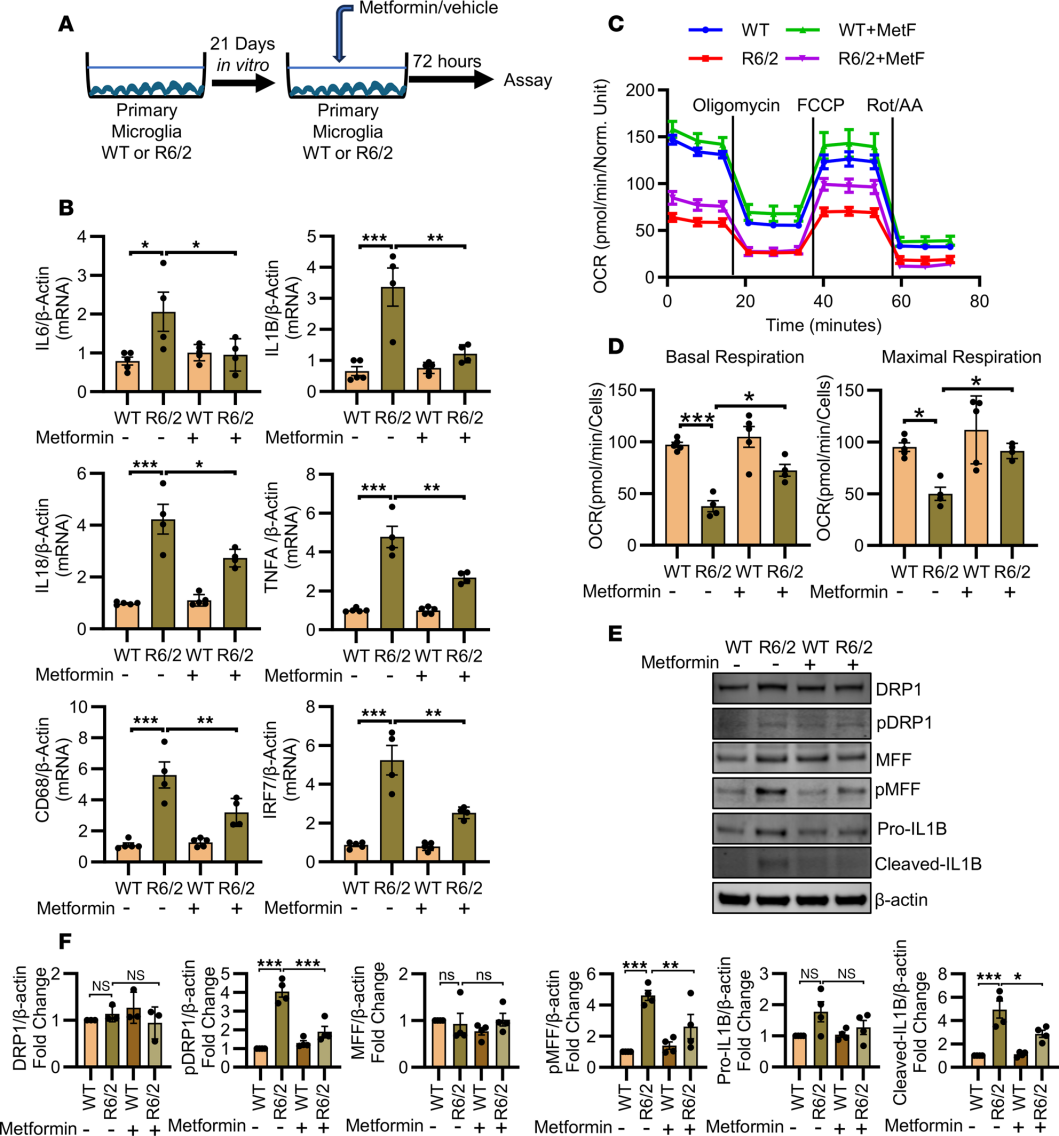
Metformin reprograms microglial metabolism via mitochondrial fission. (**A**) Schematic diagram of metformin treatment in WT and R6/2 mouse primary microglia. (**B**) qPCR analysis of proinflammatory and microglial activation marker mRNAs (*IL6*, *IL1B*, *IL18*, *TNFA*, *CD68*, and *IRF7*) in primary microglial cells isolated from WT (*n* = 5) and R6/2 (*n* = 4) mouse brains following treatment with metformin or vehicle. (**C**) Real-time oxygen consumption rate (OCR) measurements in WT and R6/2 primary microglia treated with or without metformin (25 μM) for 72 hours. Mitochondrial stress test was performed using sequential injections of oligomycin, FCCP, and rotenone/antimycin A (Rot/AA); OCR was normalized to nuclear staining. (**D**) Quantification of basal and maximal respiration from the same experiment. OCR was measured using a Seahorse XFe96 analyzer; WT, *n* = 5; R6/2, *n* = 4. (**E** and **F**) Representative immunoblot images (**E**) and their normalized quantification (**F**) showing expression of DRP1 (*n* = 3), phosphorylated DRP1, MFF, phosphorylated MFF, pro-IL1B, cleaved IL1B, and β-actin (*n* = 4) in primary microglia. Data in all panels are presented as mean ± SEM and were analyzed by 2-way ANOVA followed by Tukey’s test. *P < 0.05; **P < 0.01; ***P < 0.001.

Impaired metabolic regulation and microglial activation were observed in the R6/2 mouse brain, with metformin rescuing these abnormalities (Figures 3 and 4). We then performed real-time metabolic assessments in primary microglia isolated from WT and R6/2 mice (Figure 6A). Primary microglia were maintained in vitro for 21 days to acquire adult-like phenotypes (57, 58). Similar to the in vivo findings, primary microglia from R6/2 mice exhibited significant upregulation of proinflammatory cytokines (*IL6*, *IL1B*, and *IL18*) and markers of microglial activation (*TNFA*, *CD68*, and *IRF7*) (Figure 6B). Treatment with metformin for 72 hours significantly reduced the expression of these proinflammatory markers (Figure 6B). Next, we evaluated real-time bioenergetics in primary microglia from R6/2 mice to assess the effect of metformin on mitochondrial respiration. Using an extracellular flux analyzer, we measured the oxygen consumption rate, a key indicator of mitochondrial respiration (Figure 6B). We found that basal respiration was significantly reduced in R6/2 primary microglia compared with WT controls (Figure 6, C and D). Notably, metformin treatment enhanced basal respiration in R6/2 microglia (Figure 6, C and D), with no effect observed in WT microglia (Figure 6, C and D). Maximal respiratory capacity was also decreased in R6/2 microglia compared with WT, which was significantly improved by metformin (Figure 6, C and D). However, spare respiratory capacity remained unchanged in R6/2 microglia and was unaffected by metformin treatment (Supplemental Figure 2). These findings demonstrate that R6/2 microglia exhibit metabolic impairments, which are reversed by metformin, highlighting the potential of metformin to reprogram microglial metabolism. It is of interest to note that HD microglia express mutant huntingtin, suggesting that in addition to an indirect reaction from neuronal toxicity, additional mitochondrial stress may take place endogenously in microglia mediated by mutant huntingtin (17–20).

Further, we investigated the effects of metformin on mitochondrial fission in primary microglia derived from R6/2 mice, because altered mitochondrial dynamics contribute to microglial dysfunction and neuroinflammation in HD (59, 60). Metformin treatment reduced dynamin-related protein 1 phosphorylation (p-DRP1) at Ser616, a modification associated with increased mitochondrial fragmentation in HD (Figure 6, E and F) (59). Similarly, mitochondrial fission factor phosphorylation (p-MFF) was decreased following metformin treatment in HD microglia, indicating a coordinated downregulation of fission machinery (Figure 6, E and F). These changes were complemented by a reduction of IL1B accumulation (Figure 6, E and F), which suggests that mitochondrial fission is associated with metabolic reprogramming and microglial activation in R6/2 microglia. In parallel, metformin treatment led to a significant reduction in *IL1B* mRNA accumulation (Figure 6B) and cleavage of pro-IL1B, generating active mature IL1B protein (Figure 6, E and F). Similarly, metformin reduced phosphorylation of DRP1 and MFF, markers of mitochondrial fission, in the R6/2 striatum (Supplemental Figure 3). This was accompanied by decreased IL1B, indicating reduced neuroinflammation (Supplemental Figure 3). Reduced IL1B level in R6/2 mice is observed with caspase-1 inhibition and minocycline-mediated neuroprotection (18, 21). Improved mitochondrial fission is associated with enhanced OXPHOS, as demonstrated by increased basal and maximal oxygen consumption rates measured via Seahorse assay (Figure 6, C and D). Together, these findings suggest that metformin promotes mitochondrial health by regulating the phosphorylation status of DRP1 and MFF. Collectively, these results demonstrate that metformin enhances mitochondrial function by modulating mitochondrial fission and simultaneously mitigates neuroinflammatory responses.

## Discussion

Microglial activation is a hallmark of neurodegenerative diseases, including HD, yet the mechanisms underlying this activation remain poorly understood. Recent studies have linked alterations in cellular metabolic pathways to the dysfunction of immune cells, including microglia (61). In this study, we demonstrate that metformin regulates mtDNA release, proinflammatory gene expression, microglial activation, and metabolic dysregulation in R6/2 mice, with key findings corroborated with human HD samples. Our findings highlight the potential of metformin as a therapeutic agent for modulating neuroinflammation and metabolic dysfunction in HD.

Previously, we identified mtDNA release as a regulatory switch for neuroinflammation (46). Consistent with this, we observed elevated mtDNA release in the cortex and striatum of HD R6/2 mice, which was rescued by metformin. Increased mtDNA release promotes cGAS/STING signaling, a pathway that triggers inflammatory responses. Elevated levels of proinflammatory cytokines have been reported in the central nervous system and plasma of HD patients and in other neurodegenerative diseases (5, 25, 37, 62–66). In line with these and our earlier findings that inflammation in R6/2 mice can be attenuated by caspase-1 inhibition and minocycline treatment (18, 21, 67, 68), the present study further demonstrates that metformin similarly reduces and attenuates the expression of proinflammatory cytokines and microglial activation markers in the R6/2 brain. This suggests that metformin’s antiinflammatory effects may be mediated in part by its ability to regulate mtDNA release and downstream signaling pathways, thereby modulating neuroinflammation in HD.

Activated microglia, which exhibit a proinflammatory phenotype, are known to contribute to neuroinflammation and synaptic degeneration in HD (69). Our data indicate that metformin treatment reduces the microglial activation in R6/2 mice, as evidenced by the downregulation of microglial activation markers, such as CD16, CD68, CD86, etc. Additionally, we observed that metformin significantly increased the ramification of microglial cells, which is a characteristic of the quiescent microglia. This effect was reflected in the increased area, process length, number of nodes, and number of endings of microglial processes in metformin-treated R6/2 mice. These morphological changes suggest that metformin not only inhibits proinflammatory microglial activation but also restores the quiescent phenotype (70, 71). It is of interest that HD microglia express mutant huntingtin. This, and the fact that isolated R6/2 microglia demonstrate aberrant metabolism, suggest that the HD microglial phenotype is likely mediated by direct endogenous mutant huntingtin toxicity, superimposed upon a pathological neuronal milieu in HD (67).

The metabolic flexibility of immune cells, including microglia, enables them to adapt to different microenvironments and modulate immune responses (1–4). In the context of HD, metabolic reprogramming plays a crucial role in microglial activation and inflammation. Our study found that glycolytic enzymes were upregulated in the cortex and striatum of HD patients and striatum of R6/2 mice, indicating a shift toward glycolysis in response to neuroinflammation. In contrast, PDH, which catalyzes the conversion of pyruvate to acetyl-CoA for OXPHOS, was inhibited in HD samples. PDH inhibition suggests that energy production in HD reflects a shift toward glycolysis rather than OXPHOS at the level of striatal tissue. Because these measurements were performed in whole striatal homogenates, the observed metabolic changes likely represent combined contributions from all brain cell types. Metformin treatment rescued the inhibition of PDH in R6/2 striatum, restoring its activity to levels comparable to those in WT mice. PDH activity is regulated by pyruvate dehydrogenase kinase (PDHK), which phosphorylates and inactivates PDH, and by pyruvate dehydrogenase phosphatase, which dephosphorylates and activates PDH. We found that PDHK expression was reduced in R6/2 striatal tissue and normalized by metformin, suggesting that metformin may influence PDH activity through modulation of PDHK within the broader striatal cellular environment. Although these tissue-level findings are consistent with a shift toward enhanced OXPHOS and reduced glycolytic flux, any direct link to microglial metabolic activation remains speculative without cell type–specific analyses. Importantly, our independent measurements of mitochondrial respiration in isolated R6/2 microglia treated with metformin support this possibility, but further microglia-specific metabolic studies will be required to establish a definitive mechanism.

Furthermore, metabolic reprogramming in activated microglia leads to the generation of ROS, which serve as secondary messengers in inflammatory signaling pathways (72, 73). ROS production, through NADPH oxidases, can activate the *MAPK* and *NFkB* pathways, leading to the increased synthesis of proinflammatory cytokines (74–76). By restoring PDH activity and promoting OXPHOS, metformin treatment likely reduces ROS production and mitigates the inflammatory response in HD microglia. Metformin enhances mitochondrial function and reduces neuroinflammation by regulating key components of the mitochondrial fission machinery and suppressing proinflammatory signaling. Specifically, metformin decreased the phosphorylation of DRP1 and MFF, leading to improved OXPHOS (59, 77). Modulation of these mitochondrial dynamics likely contributes to the observed reduction in proinflammatory cytokine expression, including IL-1β, suggesting that mitochondrial dynamics are closely linked to the metabolic reprogramming and microglial activation in HD. These results align with emerging evidence that mitochondrial dysfunction and inflammation are interconnected drivers of neurodegenerative pathology (78, 79). By simultaneously targeting mitochondrial fission and inflammatory signaling, metformin may offer a dual therapeutic strategy to protect against neurodegeneration. An ongoing clinical trial testing the therapeutic effects of metformin on cognitive decline in HD is a promising step toward evaluating its efficacy in humans. Our findings are consistent with previous studies identifying metabolic reprogramming as a critical event in microglial activation and neuroinflammation (80). Notably, metformin’s ability to normalize metabolic dysfunction and reduce microglial activation suggests that it may be a promising therapeutic strategy for HD.

**Figure 7:**
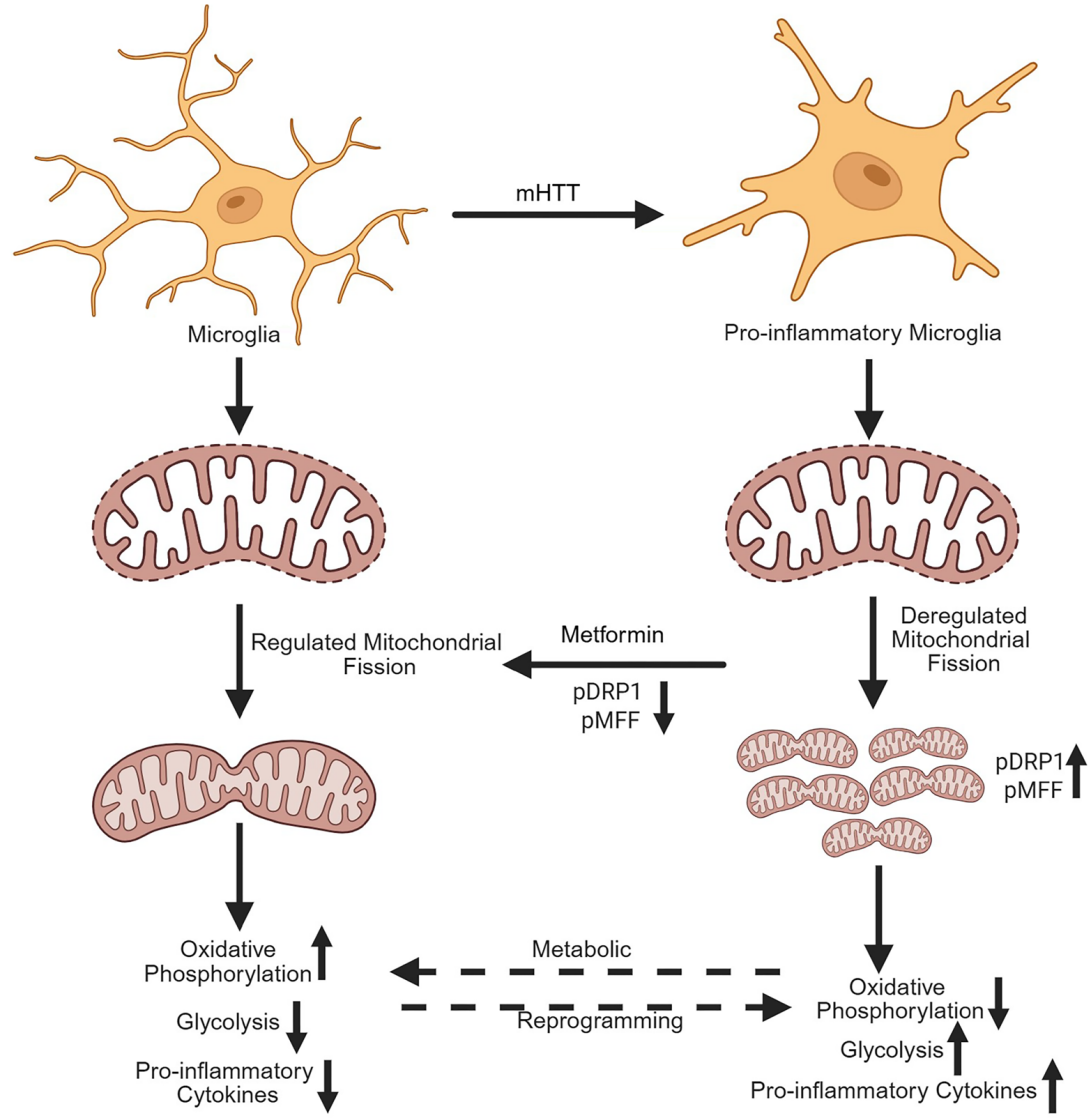
Graphical representation of metabolic reprogramming and microglial activation. Figure created with BioRender (biorender.com) under a University of Pittsburgh license.

In conclusion, metabolic reprogramming plays a pivotal role in the microglial activation and function of microglia. During activation, microglia undergo significant metabolic shifts to meet the energy demands of their functional states (Figure 7). Proinflammatory activation is typically characterized by enhanced glycolysis, providing rapid energy for immediate immune responses. Thus, our study provides compelling evidence that metabolic reprogramming plays a key role in microglial activation and neuroinflammation in HD. Metformin, through its ability to restore mitochondrial dynamics and function and regulate metabolic reprogramming, offers a novel approach to modulating these pathogenic processes. By inhibiting proinflammatory cytokine expression and promoting an antiinflammatory microglial phenotype, metformin holds significant promise as a therapeutic strategy for HD. Further studies are needed to fully explore metformin’s potential in treating HD and other neurodegenerative diseases.

## Methods

### Sex as a biological variable.

Only female R6/2 model mice and their wild-type littermate controls were used in this study. This approach was adopted to minimize variability associated with sex-specific behavioral and physiological differences, as male R6/2 mice often display increased aggression and stress-related responses and greater phenotypic variability that can confound experimental outcomes. Female mice allow for more stable group housing and more consistent assessment of disease progression and molecular endpoints. For human samples, individuals of both sexes were included in the study.

### Primary microglia culture.

Primary microglial cell cultures were derived from wild-type (WT) and R6/2 transgenic mouse pups using a previously published protocol (81). Briefly, newborn pups were harvested from breeding cages and placed on a 37°C heating plate to maintain body temperature. After decapitation, the cortices were carefully dissected under a dissection microscope. The dissected tissues were washed 3 times in dissection medium and incubated with trypsin for 15 minutes at 37°C. After incubation, the tissues were centrifuged, and the resulting cell pellets were collected. DNase was added to digest DNA released from dead cells. The pellet was then triturated with 5 mL of warm culture medium using a 1 mL pipette tip. Cells were counted and subsequently plated into T-75 flasks. The culture medium was changed the following day to remove dead cells and debris. Within 5–7 days, astrocytes formed a confluent layer at the bottom of the flask, while microglia and some oligodendrocytes grew on top of the astrocyte layer. Microglial cells were harvested by vigorous tapping of the flasks, and the detached floating cells were collected in conditioned culture medium.

### Murine tissues and animal treatment.

All mouse tissues were collected in accordance with the NIH *Guide for the Care and Use of Laboratory Animals* (National Academies Press, 2011). Animal protocols were approved by the University of Pittsburgh’s Institutional Animal Care and Use Committee (protocol 20128492). Mice were maintained on a 12-hour light/12-hour dark cycle with ad libitum access to food and water. R6/2 transgenic mice, carrying the promoter sequence and exon 1 of a mutant human *HTT* gene with approximately 160 CAG repeats, were obtained from The Jackson Laboratory (stock 002810). A breeding colony was established by mating of R6/2 males with B6CBAF1 females (The Jackson Laboratory). CAG repeat length was determined for each mouse in the colony, and only mice with 160 ± 15 CAG repeats were used in experiments. The study used 6-week-old R6/2 mice, which were treated with metformin for 4 weeks. Metformin (200 mg/kg body weight) was administered intraperitoneally 5 days per week for 4 weeks, as previously described (82).

### Human tissues.

Cortex grade IV and striatum samples of grade II HD patients, and control patients’ samples, were obtained from the New York Brain Bank at Columbia University (New York, New York, USA). Clinical information of the patients and subjects is given in Supplemental Table 4.

### Immunofluorescence.

Brains of mice injected with metformin for 4 weeks were perfused with cold PBS and 4% paraformaldehyde. Brains were stored in 4% paraformaldehyde. Sections of 20 μm thickness were sliced and stored in PBS at 4°C. Sections were washed 3 times (10 minutes each time) with phosphate-buffered saline with tween-20 and blocked with 10% donkey serum in PBS for 1 hour at room temperature. After blocking, sections were incubated with the indicated primary and secondary antibodies. Samples were imaged with an Olympus IX-81-DSU in confocal mode.

### Cytosolic mtDNA quantification.

For measurement of mtDNA in cytosol cortex and striatum, tissues were homogenized in IM buffer (5 mM HEPES-Tris [pH 7.4], 225 mM sucrose, 75 mM mannitol, and 1 mM EGTA) and divided into 2 equal parts. One part was then centrifuged at 1,300*g* for 3 minutes, and the supernatant was again spun at 20,000*g* for 10 minutes. The resultant supernatant was collected as the cytosolic fraction. Cytosolic fraction (200 μL) and corresponding total tissue or cellular homogenate were used to isolate cytosolic and total DNA, respectively. The copy number of mtDNA-encoding cytochrome *c* oxidase 1 (mt-CO1), mt-Dloop1, and mt-Dloop3 was measured by quantitative PCR (qPCR) with the same volume of DNA solution. Normalization to the nuclear genome was performed with DNA isolated from tissue homogenate using β-actin.

### Real-time PCR.

Total RNA was isolated using the RNeasy Plus kit (QIAGEN) as described by the manufacturer and quantified using an ND-1000 spectrophotometer (Thermo Fisher Scientific). cDNA was prepared using 1,000 ng total RNA by reverse transcription PCR using a high-capacity cDNA reverse transcription kit (Applied Biosystems) according to the manufacturer’s instructions. qPCR was performed on cDNA using SYBR Green chemistry. qPCR was performed on a Bio-Rad CFX Touch PCR system and an Applied Biosystems QuantStudio 5 PCR System using Applied Biosystems PowerUp SYBR Green master mix. For SYBR primer pair sequences, see Supplemental Table 5. Fold change in expression was calculated by the ΔΔCt method using β-actin as an endogenous control for mRNA expression. All fold changes were expressed as normalized to untreated control. Cytosolic mtDNA was quantified as described in our previous publication (46). All details regarding primers used in real-time PCR are given in Supplemental Table 5.

### Immunoblotting.

Immunoblotting was performed as described previously (46). In brief, cell or tissue lysates were cleared by centrifugation at 20,000*g*, equal amounts of protein were separated on Novex 4%–12% gradient polyacrylamide gels (Invitrogen), and proteins were transferred overnight onto 0.45 μm polyvinylidene difluoride membranes. The membranes were then incubated with the indicated primary antibodies overnight at 4°C followed by incubation with secondary antibodies (Li-Cor) for 1 hour at room temperature and thereafter analyzed using the Odyssey CLx Infrared Imaging System (Li-Cor). Band intensities were quantified using Image Studio (Li-Cor). All details regarding the antibodies used in immunoblotting are given in Supplemental Table 6.

### Extracellular flux analysis.

Extracellular flux (XF) analysis was performed as described in our previous publication (83). Briefly, cells were seeded in an XFe 96-well cell culture microplate (Seahorse Bioscience) in sextuplicate at 1 × 10^4^ cells per well in 100 μL growth medium and then incubated at 37°C in 5% CO_2_. Cells were seeded just before the day of the experiment. To initiate assays, the growth medium was removed from each well and replaced with 180 μL of assay medium (DMEM Seahorse assay medium with 10 μM glucose and 5 μM pyruvate) prewarmed to 37°C. The cells were incubated at 37°C for 45 minutes to allow the temperature and pH of the medium to reach equilibrium before the first rate measurement. Before each rate measurement, the XFe96 Analyzer gently mixed the assay medium in each well for 3 minutes to allow the oxygen partial pressure to reach equilibrium. After mixing, the oxygen consumption rate (OCR) and ECAR were measured simultaneously for 3 minutes to establish a baseline rate. The assay medium was then gently mixed again for 3 minutes between each rate measurement to restore normal oxygen tension and pH in the microenvironment surrounding the cells. After the baseline measurement, 20–25 μL of a testing agent prepared in assay medium was then injected into each well to reach the desired final working concentration. This was followed by mixing for 3 minutes to expedite compound exposure to cellular proteins, after which OCR and ECAR measurements were made. Generally, 3 baseline rates and 3 response rates (after compound addition) were measured. The values of OCR and ECAR reflect both the metabolic activities of the cells and the number of cells being measured. Typically, at the end of each assay, cell number was normalized using nuclear stain fluorescence intensity values.

### Statistics.

Statistical analyses were performed with GraphPad Prism 10.2.2. Data were obtained from at least 3 independent experiments and are expressed as mean ± SEM unless otherwise specified. The 2-tailed Student’s *t* test for parametric data and 2-tailed paired *t* tests were used for experiments with multiple samples from the same source. One-way ANOVA or 2-way ANOVA followed by Tukey’s test was used for analysis of more than 2 groups as specified in each figure legend. *P* values less than 0.05 were considered statistically significant (*P < 0.05; **P < 0.01; ***P < 0.001).

### Study approval.

All animal experiments were approved by the University of Pittsburgh Institutional Animal Care and Use Committee (approved protocol 20128492) and conducted in accordance with institutional and national guidelines for the care and use of laboratory animals.

### Data availability.

All data supporting the findings of this study are included in the article and its supplemental materials. Source data underlying all figures are provided in the Supporting Data Values Excel file.

## Author contributions

AJ, DLC, and RMF designed experiments. AJ, ACM, OSA, SS, TS, and CSC generated data. XW bred and maintained the R6/2 colony. AJ, DLC, and RMF wrote the manuscript. AJ and RMF conceived the initial study.

## Conflict of interest

RMF is the founder of Helexva, and DLC is a scientific consultant for Helexva. Helexva is focused on developing small-molecule treatments for Huntington’s disease. No funding was provided by Helexva for this project.

## Funding support

This work is the result of NIH funding, in whole or in part, and is subject to the NIH Public Access Policy. Through acceptance of this federal funding, the NIH has been given a right to make the work publicly available in PubMed Central.

- NIH grant R01NS100743 (to RMF)
- Pittsburgh Foundation Walter L. Copeland Fund (to AJ)
- Huntington’s Disease Society of America Donald A. King Fellowship (to ACM)

## Supplementary Material

Supplemental data

Unedited blot and gel images

Supporting data values

## Version 1 04/02/2026

In-Press Preview

## Version 2 05/22/2026

Electronic publication
