# Supplementary material for: Metabolic reprogramming is critical to microglial activation in Huntington’s disease
Source: JCI Insight. 2026 Apr 2;11(10):e201466. doi: 10.1172/jci.insight.201466 (PMC13232724; doi:10.1172/jci.insight.201466)
Supplement: Supplemental data [file jciinsight-11-201466-s149.pdf]

Supplemental Information

	WT<br>Vehicle	R6/2<br>Vehicle	WT<br>Metformin	R6/2<br>Metformin	pValue<br>WTvsR6/2	pValue<br>R6/2 Metformin vs R6/2 Metformin
cGAS	1 ± 0.28	2.1 ± 0.9	0.98 ± 0.21	0.91 ± 0.44	0.0169	0.008
STING	1 ± 0.36	1.73 ± 0.2	1.64 ± 0.52	1.08 ± 0.47	0.1482	0.2062
IRF3	1 ± 0.38	2 ± 0.06	1.4 ± 0.33	1.31 ± 0.54	0.0348	0.1321
IL-6	1 ± 0.38	2.43 ± 0.74	1.04 ± 0.22	1.48 ± 0.24	0.0036	0.0335
IL-1β	1 ± 0.34	3.7 ± 1.46	1.12 ± 0.35	1.31 ± 0.22	0.0007	0.0012
IL-18	1 ± 0.05	2.58 ± 0.1	1.2 ± 0.48	0.9 ± 0.19	<0.0001	<0.0001
IFNα	1 ± 0.18	4.71 ± 2.75	1.43 ± 0.4	1.87 ± 0.92	0.0115	0.0425
IFNβ	1 ± 0.5	4.25 ± 2.55	2.13 ± 1.31	1.69 ± 0.9	0.0122	0.0281
B2M	1 ± 0.07	1.83 ± 0.51	1.16 ± 0.18	1.22 ± 0.34	0.0007	0.0019
OAS1	1 ± 0.1	3.99 ± 0.49	1.22 ± 0.42	2.53 ± 0.38	<0.0001	0.0013
Clec7A	1 ± 0.1	2.26 ± 0.32	1.05 ± 0.53	0.66 ± 0.46	0.0041	0.0009
IFI27L2A	1 ± 0.22	3.59 ± 1.22	1.19 ± 0.45	2.29 ± 0.41	0.0015	0.068
SPP1	1 ± 0.09	3.74 ± 1.14	0.69 ± 0.13	1.97 ± 0.54	<0.0001	0.0016
AXL	1 ± 0.17	0.67 ± 0.07	0.94 ± 0.15	0.62 ± 0.12	0.0654	0.9166
TREM2	1 ± 0.24	0.76 ± 0.17	1.08 ± 0.24	0.95 ± 0.33	0.5306	0.6475
CXCL10	1 ± 0.54	1.45 ± 0.48	0.58 ± 0.28	1.81 ± 0.35	0.5044	0.642
IRF7	1 ± 0.45	0.7 ± 0.3	1.25 ± 0.6	1.34 ± 0.47	0.6917	0.2489
CST7	1 ± 0.21	1.31 ± 0.39	1.28 ± 0.46	1.42 ± 0.44	0.5015	0.9107
AIF1	1 ± 0.34	1.02 ± 0.23	1.09 ± 0.27	0.87 ± 0.11	0.9851	0.6196
PSD95	1 ± 0.07	0.57 ± 0.07	0.76 ± 0.26	0.72 ± 0.1	0.0079	0.3116
Homer1	1 ± 0.12	0.58 ± 0.08	0.89 ± 0.18	0.77 ± 0.14	0.0031	0.1959
SYP	1 ± 0.03	1.16 ± 0.15	1 ± 0.13	1.28 ± 0.28	0.5607	0.7352
SYN1	1 ± 0.17	1.13 ± 0.16	0.96 ± 0.21	1 ± 0.23	0.7672	0.7695
C1qB	1 ± 0.44	2.27 ± 0.2	1.33 ± 0.13	1.42 ± 0.36	0.0036	0.0238
C4B	1 ± 0.13	3.12 ± 0.36	1.14 ± 0.23	1.49 ± 0.07	<0.0001	0.0002
C3	1 ± 0.23	1.02 ± 0.04	0.79 ± 0.15	0.71 ± 0.26	0.9836	0.1769

Supplemental Table 1. Quantitative gene expression analysis in the cortex of WT and R6/2 mouse brain. The table shows relative mRNA expression levels of analyzed genes expressed as fold change (mean ± SD). Gene expression was measured by quantitative real-time PCR (qPCR) and calculated using the ΔΔCt method with β-actin as the endogenous normalization control, n=3-5. Statistical significance was determined by two-way ANOVA with comparisons between WT vs R6/2 and R6/2 vs R6/2 ± metformin. Corresponding *P* values are indicated.

	WT<br>Vehicle	R6/2<br>Vehicle	WT<br>Metformin	R6/2<br>Metformin	pValue<br>WTvsR6/2	pValue<br>R6/2 Metformin vs R6/2 Metformin
cGAS	1 ± 0.46	2.91 ± 0.52	1.08 ± 0.42	1.3 ± 0.34	0.0004	0.0009
STING	1 ± 0.21	3.54 ± 0.73	1.13 ± 0.25	1.74 ± 0.5	<0.0001	0.0002
IRF3	1 ± 0.3	6.03 ± 2.08	0.84 ± 0.35	2.18 ± 0.77	0.0001	0.0006
IL-6	1 ± 0.21	3.85 ± 1.5	1.32 ± 0.5	1.64 ± 0.25	0.0008	0.0035
IL-1β	1 ± 0.35	3.39 ± 0.13	1.09 ± 0.11	2.46 ± 0.45	0.0001	0.019
IL-18	1 ± 0.11	5.42 ± 0.42	1.23 ± 0.23	2.12 ± 1.19	<0.0001	0.0001
IFNα	1 ± 0.15	4.34 ± 1.39	1.22 ± 0.44	1.89 ± 0.84	0.0002	0.0026
IFNβ	1 ± 0.33	8.68 ± 3.72	2.15 ± 0.74	2.76 ± 0.64	0.0004	0.002
B2M	1 ± 0.1	2.08 ± 0.45	1.06 ± 0.28	1.57 ± 0.34	0.0003	0.0187
OAS1	1 ± 0.34	9.57 ± 3.89	1.28 ± 0.63	3.14 ± 1.75	0.0001	0.001
Clec7A	1 ± 0.05	2.6 ± 0.91	0.73 ± 0.32	1.4 ± 0.29	0.0039	0.0181
IFI27L2A	1 ± 0.19	12.13 ± 2.32	1.98 ± 0.98	4.19 ± 1.23	<0.0001	<0.0001
SPP1	1 ± 0.41	4.49 ± 1.7	1.55 ± 1.15	1.78 ± 0.7	0.0059	0.0214
AXL	1 ± 0.31	0.46 ± 0.06	1.01 ± 0.84	0.62 ± 0.14	0.3409	0.8958
TREM2	1 ± 0.27	1.58 ± 0.75	0.89 ± 0.16	0.93 ± 0.15	0.2974	0.23
CXCL10	1 ± 0.58	14.46 ± 9.43	2.11 ± 1.05	3.91 ± 1.65	0.0036	0.0144
IRF7	1 ± 0.23	10.16 ± 3.58	1.29 ± 0.32	5.22 ± 0.93	<0.0001	0.0047
CST7	1 ± 0.37	7.14 ± 5.3	0.95 ± 0.49	2.04 ± 0.46	0.0643	0.119
PSD95	1 ± 0.2	0.48 ± 0.09	1.06 ± 0.77	0.59 ± 0.14	0.3038	0.9346
Homer1	1 ± 0.35	0.31 ± 0.03	0.62 ± 0.3	0.42 ± 0.12	0.0225	0.829
SYP	1 ± 0.36	0.68 ± 0.1	0.99 ± 0.88	0.85 ± 0.16	0.7007	0.897
SYN1	1 ± 0.41	0.27 ± 0.06	0.57 ± 0.36	0.69 ± 0.13	0.0254	0.1763
C1qA	1 ± 0.21	1.95 ± 0.42	1.28 ± 0.29	1.39 ± 0.32	0.0219	0.1446
C1qB	1 ± 0.34	2.63 ± 0.51	1.57 ± 0.35	1.46 ± 0.22	0.0049	0.0232

C4B	1 ± 0.38	4.1 ± 2.27	1.67 ± 0.14	1.73 ± 0.16	0.0261	0.0736
C3	1 ± 1.13	1.3 ± 0.03	1.04 ± 0.55	0.72 ± 0.15	0.8592	0.5843

Supplemental Table 2. Quantitative gene expression analysis in the striatum of WT and R6/2 mouse brain. The table shows relative mRNA expression levels of analyzed genes expressed as fold change (mean ± SD). Gene expression was measured by quantitative real-time PCR (qPCR) and calculated using the ΔΔCt method with β-actin as the endogenous normalization control, n=3-5. Statistical significance was determined by two-way ANOVA with comparisons between WT vs R6/2 and R6/2 vs R6/2 ± metformin. Corresponding *P* values are indicated.

	WT Vehicle	R6/2 Vehicle	WT Metformin	R6/2 Metformin	pValue WTvsR6/2	pValue R6/2 Metformin vs R6/2 Metformin
GLUT1	1 ± 0.18	1.76 ± 0.39	0.88 ± 0.12	1.23 ± 0.07	0.0032	0.0248
GLUT3	1 ± 0.14	1.33 ± 0.45	0.97 ± 0.12	1.08 ± 0.11	0.2222	0.3905
HK1	1 ± 0.06	1.67 ± 0.18	0.94 ± 0.15	1.15 ± 0.13	0.0003	0.0015
HK2	1 ± 0.16	1.84 ± 0.28	1.06 ± 0.08	1.43 ± 0.1	<0.0001	0.0095
PFKM	1 ± 0.13	0.91 ± 0.38	0.89 ± 0.09	0.98 ± 0.1	0.8327	0.9043
PFKP	1 ± 0.12	1.43 ± 0.2	0.88 ± 0.14	1.05 ± 0.05	0.0022	0.0051
ALDOA	1 ± 0.12	1.23 ± 0.22	0.92 ± 0.08	1.14 ± 0.09	0.0977	0.6246
TPI	1 ± 0.25	1.39 ± 0.13	1.17 ± 0.16	1.21 ± 0.09	0.0253	0.3536
GAPDH	1 ± 0.18	1.54 ± 0.16	1.08 ± 0.13	1.21 ± 0.1	0.0021	0.0332
PGK1	1 ± 0.27	1.38 ± 0.15	1.2 ± 0.11	1.53 ± 0.04	0.0284	0.4623
PGAM	1 ± 0.24	1.59 ± 0.15	1.2 ± 0.12	1.08 ± 0.2	0.0021	0.0054
ENO1	1 ± 0.22	1.53 ± 0.16	1.03 ± 0.2	1.35 ± 0.08	0.0001	0.0771
ENO2	1 ± 0.13	1.59 ± 0.17	1.43 ± 0.21	1.41 ± 0.05	<0.0001	0.0802
PKM	1 ± 0.27	1.67 ± 0.13	1.27 ± 0.1	1.2 ± 0.22	0.0033	0.0248
LDHA	1 ± 0.17	1.83 ± 0.13	0.91 ± 0.22	0.91 ± 0.45	0.0054	0.0029
LDHB	1 ± 0.33	1.73 ± 0.61	0.94 ± 0.34	0.63 ± 0.16	0.0459	0.0052
PDK1	1 ± 0.29	2.33 ± 0.65	1.48 ± 0.89	2.47 ± 1.84	0.155	0.9735
PDK2	1 ± 0.77	1.07 ± 0.3	0.94 ± 0.47	1.22 ± 0.5	0.9779	0.9148
PDK3	1 ± 0.52	1.52 ± 0.39	0.87 ± 0.39	1.35 ± 0.31	0.2384	0.8356
PDK4	1 ± 0.28	1.92 ± 0.46	0.82 ± 0.36	1.28 ± 0.26	0.0084	0.0516
PDP1	1 ± 0.58	0.62 ± 0.16	1.19 ± 0.76	0.9 ± 0.12	0.5224	0.695
PDP2	1 ± 0.3	0.53 ± 0.14	1.45 ± 0.49	1.01 ± 0.31	0.17	0.1558
PDHA1	1 ± 0.43	0.61 ± 0.2	1.36 ± 0.43	1.08 ± 0.29	0.3097	0.2019
PDHB	1 ± 0.37	0.65 ± 0.26	1.11 ± 0.08	1.01 ± 0.31	0.1553	0.1393

PDHX	1 ± 0.37	0.58 ± 0.2	1.48 ± 0.43	1.2 ± 0.34	0.2296	0.0635
CS	1 ± 0.35	0.5 ± 0.16	1.24 ± 0.38	0.94 ± 0.28	0.0924	0.1387
ACO2	1 ± 0.34	0.35 ± 0.1	1.2 ± 0.39	0.73 ± 0.08	0.0199	0.1754
IDH2	1 ± 0.24	0.61 ± 0.21	1.44 ± 0.62	0.98 ± 0.18	0.3234	0.3543
OGDH	1 ± 0.16	1.05 ± 0.17	1.13 ± 0.31	0.75 ± 0.11	0.9455	0.1513
SUCLA1	1 ± 0.23	1.77 ± 0.12	0.94 ± 0.09	1.05 ± 0.23	0.001	0.0015
SUCLA2	1 ± 0.19	1.61 ± 0.36	0.96 ± 0.22	1.2 ± 0.48	0.0916	0.2945
SDHA	1 ± 0.35	1.95 ± 0.72	0.76 ± 0.28	1.16 ± 0.38	0.031	0.0689
SDHB	1 ± 0.53	0.57 ± 0.07	0.8 ± 0.13	0.86 ± 0.25	0.1393	0.3687
SDHC	1 ± 0.24	1.81 ± 0.35	1.23 ± 0.1	1.32 ± 0.47	0.0232	0.1691
SDHD	1 ± 0.08	1.98 ± 0.64	1.07 ± 0.34	1.51 ± 0.37	0.0038	0.1336

Supplemental Table 3. Effect of Metformin on gene expression of metabolic pathways in the striatum of WT and R6/2 mouse brain. The table summarizes relative mRNA expression levels of genes involved in glycolysis, pyruvate-to-acetyl-CoA conversion, and the electron transport chain, expressed as fold change (mean ± SD). Gene expression was quantified by quantitative real-time PCR (qPCR) and calculated using the ΔΔCt method with β-actin as the endogenous normalization control, n=4. Statistical analysis was performed using two-way ANOVA, with comparisons between WT vs R6/2 and R6/2 vs R6/2 ± metformin. Corresponding *P* values are provided.

Type	Grade	Specimen	Age	Gender	CAG repeats	Standard brain block (SBB)(64)	Postmortem interval before frozen
Control	n.a.	T-110	62	M	N.E.	SBB7.1	N.E.
Control	n.a.	T-133	33	F	N.E.	SBB7.1	11:25
Control	n.a.	T-169	69	M	N.E.	SBB6.2	49:20
Control	n.a.	T-180	52	F	N.E.	SBB7.2	6:07
Control	n.a.	T-638	78	M	N.E.	SBB7.1	8:00
HD2	2	T-461	77	M	41/15	SBB7.2	19:28
HD2	2	T-3221	58	M	42/15	SBB7.2	85:11
HD2	2	T-4394	75	F	41/17	SBB7.2	22:50
HD2	2	T-4498	59	M	43/17	SBB6.2	32:45
HD2	2	T-4964	89	M	40/17	SBB6.0	10:35

Supplemental Table 4: Characteristics of human samples: Grade-diagnosed HD grade, Specimen-frozen tissue samples, Age-years at death, F; female, M; male, N.E; not estimated, n.a; not applicable, CAG repeats - number of CAG repeats of both alleles.

Oligonucleotide/Primers sequence	
mt-CO1	FP; 5'-GCCCCAGATATAGCATTCCC-3'
	RP; 5'-GTTCATCCTGTTCCTGCTCC-3'
mt-Dloop1	FP; 5'-AATCTACCATCCTCCGTGAAACC-3'
	RP; 5'-TCAGTTTAGCTACCCCCAAGTTTAA-3'
mt-Dloop3	FP; 5'-TCCTCCGTGAAACCAACAA-3'
	RP; 5'-AGCGAGAAGAGGGGCATT-3'
cGAS	FP; 5'-ACCGGACAAGCTAAAGAAGGTGCT-3'
	RP; 5'-GCAGCAGGCGTTCCACAACTTTAT-3'
STING	FP; 5'-GTCCTCTATAAGTCCCTAAGCATG-3'
	RP; 5'-AAGATCAACCGCAAGTACCC-3'
IRF3	FP; 5'-CACAAGGACAAGGACGGAG-3'
	RP; 5'-ATGCAGAACCACAGAGTGTAG-3'
IL-6	FP; 5'-CCACTCACCTCTTCAGAACG-3'
	RP; 5'-CATCTTTGGAAGGTTCAGGTTG-3'
IL-1β	FP; 5'-ACGGACCCCAAAAGATGAAG-3'
	RP; 5'-TTCTCCACAGCCACAATGAG-3'
IL-18	FP; 5'-GCCTCAAACCTTCCAAATCAC-3
	RP; 5'-GTTGTCTGATTCCAGGTCTCC-3'
IFN-α	FP; 5'-TCTGTGCTTTCCTGATGGTC-3'
	RP; 5'-GGTTATGAGTCTGAGGAAGGTC-3'
IFN-β	FP; 5'-CAGCCCTCTCCATCAACTATAAG-3'
	RP; 5'-TCTCCGTCATCTCCATAGGG-3'
PSD95	FP; 5'-GTGACAACCAAGAAATACCGC-3'
	RF; 5'-TTCACCTGCAACTCATATCCTG-3'
Synaptophysin	FP; 5'-AGTGCCCTCAACATCGAAG-3'
	RP; 5'-GCCACGGTGACAAAGAATTC-3'
Homer-1	FP; 5'-CAGAGCAAGTTTTCATTGGGC-3'
	RP; 5'- TGTGTTCGGGTCAATCTGG-3'
B2M	FP;5'-TGGTCTTTCTGGTGCTTGTC-3'-
	RP; 5'-GGGTGGAACTGTGTTACGTAG-3'
OAS1	FP;5'-GGGAGACAAGAGATTCAGAAGG-3'-
	RP; 5'-CCAGGCAGCTAAGACAAGAG-3'
Clec7A	FP; 5'-CTTTAGATGAGAAGGTGGCTCC-3'
	RP; 5'-GTGTCTCTTACTTCCATACCAGG-3'
IFI27L2A	FP; 5'-GCCCGACAAATCCACTCAG-3'
	RP; 5'-CCGATTAACACTCAGAACCCTC-3'
SPP1	FP; 5'-GTGATTTGCTTTTGCCTGTTTG-3'
	RP; 5'-GAGATTCTGCTTCTGAGATGGG-3'
AXL	FP; 5'-GTGGAAAGAGGTGAACTGGTAG-3'
	RP; 5'-GTCTCGTAGTTTCTCCTTCAGC-3'
TREM2	FP; 5'-GCAGGGTTATGAGTGACAGTTG-3'
	RP; 5'-GTTGAGGGCTTGGGACAG-3'
CXCL10	FP; 5'-TCAGCACCATGAACCCAAG-3'
	RP; 5'-CTATGGCCCTCATTCTCACTG-3'
IRF7	FP; 5'-TTGATCCGCATAAGGTGTACG-3'
	RP; 5'-TTCCCTATTTTCCGTGGCTG-3'
CST7	FP; 5'-CTGCCTAACTTCTGACACCC-3'
	RP; 5'-CACTCCTGGGTTATTGGTCTC-3'
AIF1	FP; 5'-CGATGATCCCAAATACAGCAATG-3'
	RP; 5'-CCCAAGTTTCTCCAGCATTC-3'
SYN1	FP; 5'-CTGACAACTTCTCTGGCTCTG-3'
	RP; 5'-AGGTAAAAGCAGTCTCGGTG-3'

C1qB	FP; 5’-GGGTTCCTGGCTCTGATG-3’
	RP; 5’-CTCCAAACTCACCAAGGTCTC-3’
C4b	FP; 5’-GAAGGAAACAGCAAAGGCAC-3’
	RP; 5’-TCGTAGTCTTCATTGGCATCC-3’
C3	FP; 5’-CTTAAGGTGAGGGTGGAACTG-3’
	RP; 5’-GGGACAATGACATACGGTACAG-3’
C1qA	FP; 5’-CTGAAGATGTCTGCCGAGC-3’
	RP; 5’-CCCCTGGGTCTCCTTTAAAAC-3’
β-Actin	FP; 5’-ACCTTCTACAATGAGCTGCG-3’
	RP; 5’-CTGGATGGCTACGTACATGG-3’

Supplemental Table 5. Sequences of qPCR primers used in this study. FP: Forward Primer, RP: Reverse Primer

Antibody	Catalogue	Vendor
IBA 1	Proteintech	81728-1-RR
CD16	Cell Signaling Technology	#80366
ASC	Cell Signaling Technology	#67824
HS1	Cell Signaling Technology	#3892
CD68	Proteintech	28058-1-AP
CD86	Proteintech	26903-1-AP
iNOS	Proteintech	22226-1-AP
β-actin	Proteintech	66009-1-Ig
Hexokinase 1	Cell Signaling Technology	#2024
Hexokinase 2	Cell Signaling Technology	#2867
PFKFB3	Cell Signaling Technology	#13045
PKM2	Cell Signaling Technology	#4053
PDHK1	Cell Signaling Technology	#3820
PFKP	Cell Signaling Technology	#8164
Aldolase A	Cell Signaling Technology	#8060
PDH	Cell Signaling Technology	#3205
LDHA	Cell Signaling Technology	#3582
DRP1	Proteintech	12957-1-AP
pDRP1	Cell Signaling Technology	#4494
MFF	Proteintech	17090-1-AP
pMFF	Proteintech	#36177
IL-1β	Proteintech	16806-1-AP

Supplemental Table 6. Information on antibodies used in this study

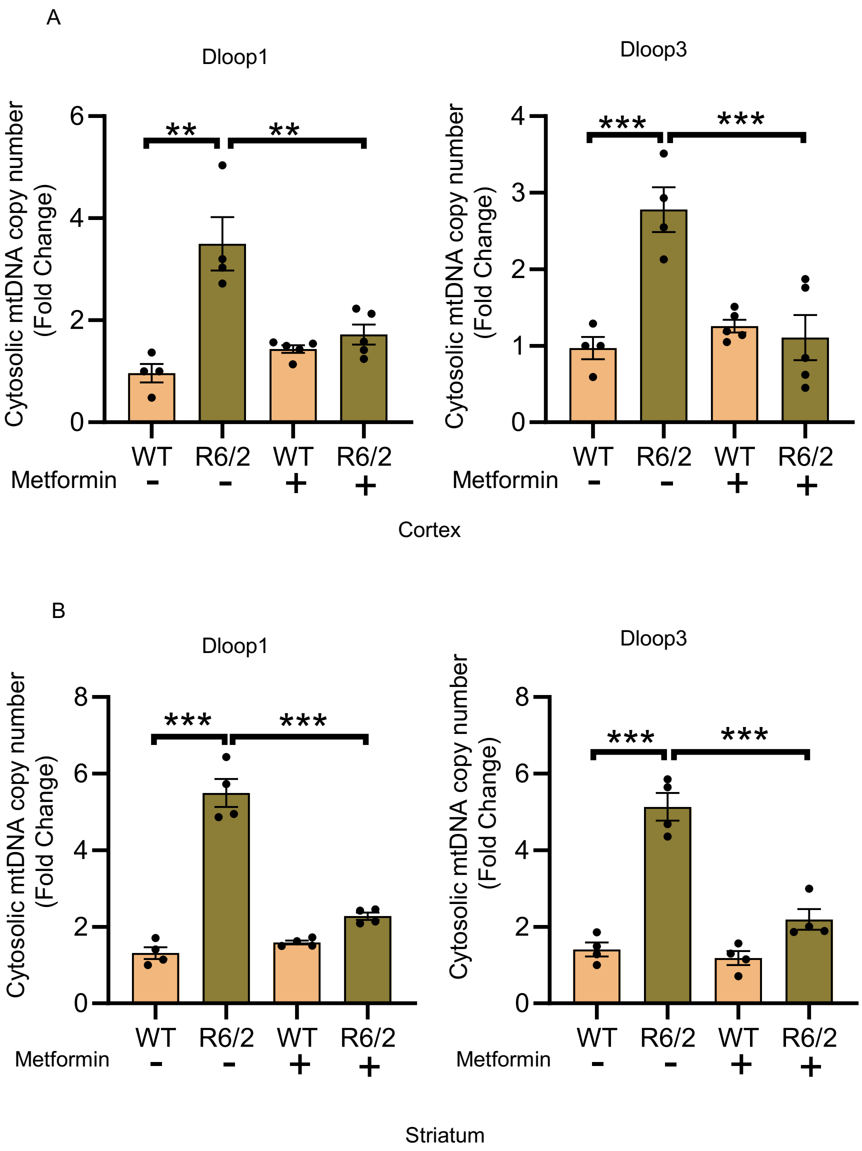

Supplemental Figure 1. Metformin regulates mtDNA release in R6/2 mice: (A) Quantification of cytosolic mtDNA in WT and R6/2 cortex treated with or without metformin (200 mg/kg b.wt) using primers against mitochondrial genes Dloop1 and Dloop3 in R6/2 cortex, n=4 in vehicle group, n=5 metformin group and (B) striatum. Data is represented as ±SEM. Individual data points in the graph represent an independent biological repeat, n=4. Data was analyzed by two-way ANOVA followed by Tukey's test. **p < 0.01; ***p < 0.001.

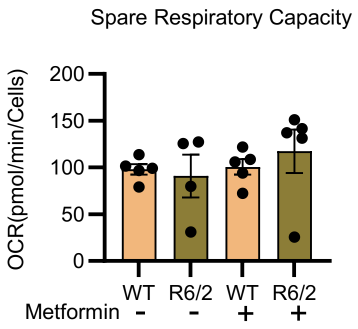

Supplemental Figure 2. Metformin reprograms microglial metabolism. Quantification of spare respiratory capacity in WT and R6/2 primary microglia treated with or without metformin (25 μM) for 72 hours by Real-time oxygen consumption rate (OCR) experiment (Figure 6c), n=5. OCR was measured using a Seahorse XF96e analyzer. Data are presented as mean ± SEM and analyzed by two-way ANOVA followed by Tukey's test.

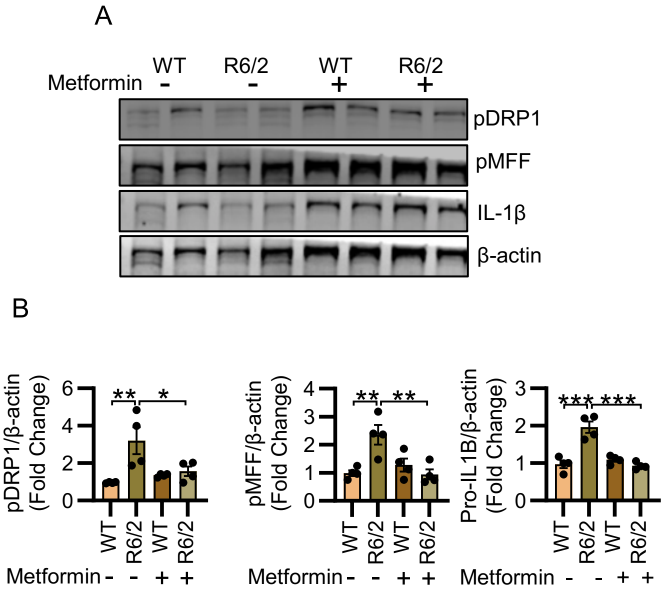

Supplemental Figure 3. Metformin modulates mitochondrial fission in R6/2 striatum (A) Representative immunoblot images of pMFF, IL1β and β-actin and their (B) normalized expression level in in striatum of WT and R6/2 mice treated with metformin or vehicle. N=4. Data is represented as ±SEM. Individual data points in the graph represent an independent biological repeat. Data was analyzed by two-way ANOVA followed by Tukey's test. *$p < 0.05$; **$p < 0.01$; ***$p < 0.001$; ns, not significant.
