# Supplementary figures and images for: Metabolic reprogramming is critical to microglial activation in Huntington’s disease
Source: JCI Insight. 2026 Apr 2;11(10):e201466. doi: 10.1172/jci.insight.201466 (PMC13232724; doi:10.1172/jci.insight.201466)

Figure 2 C

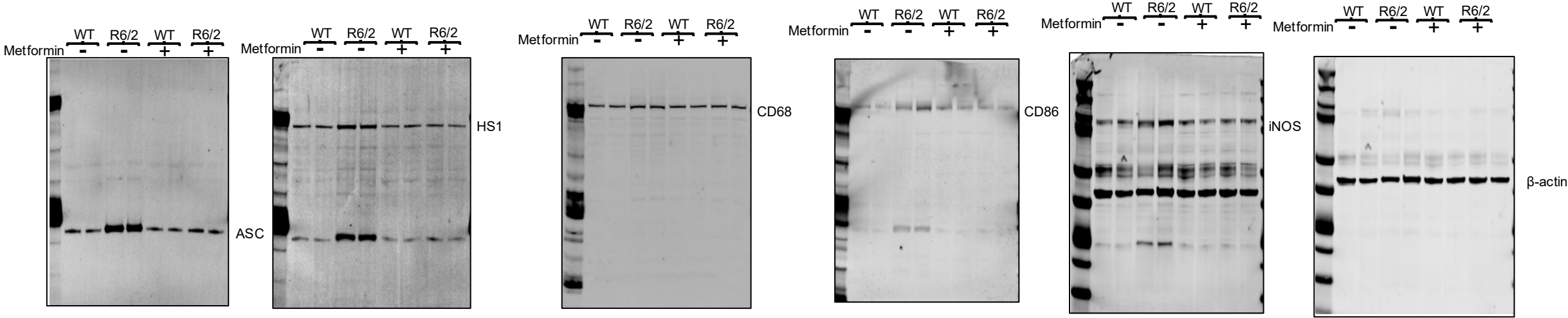

Figure 4E

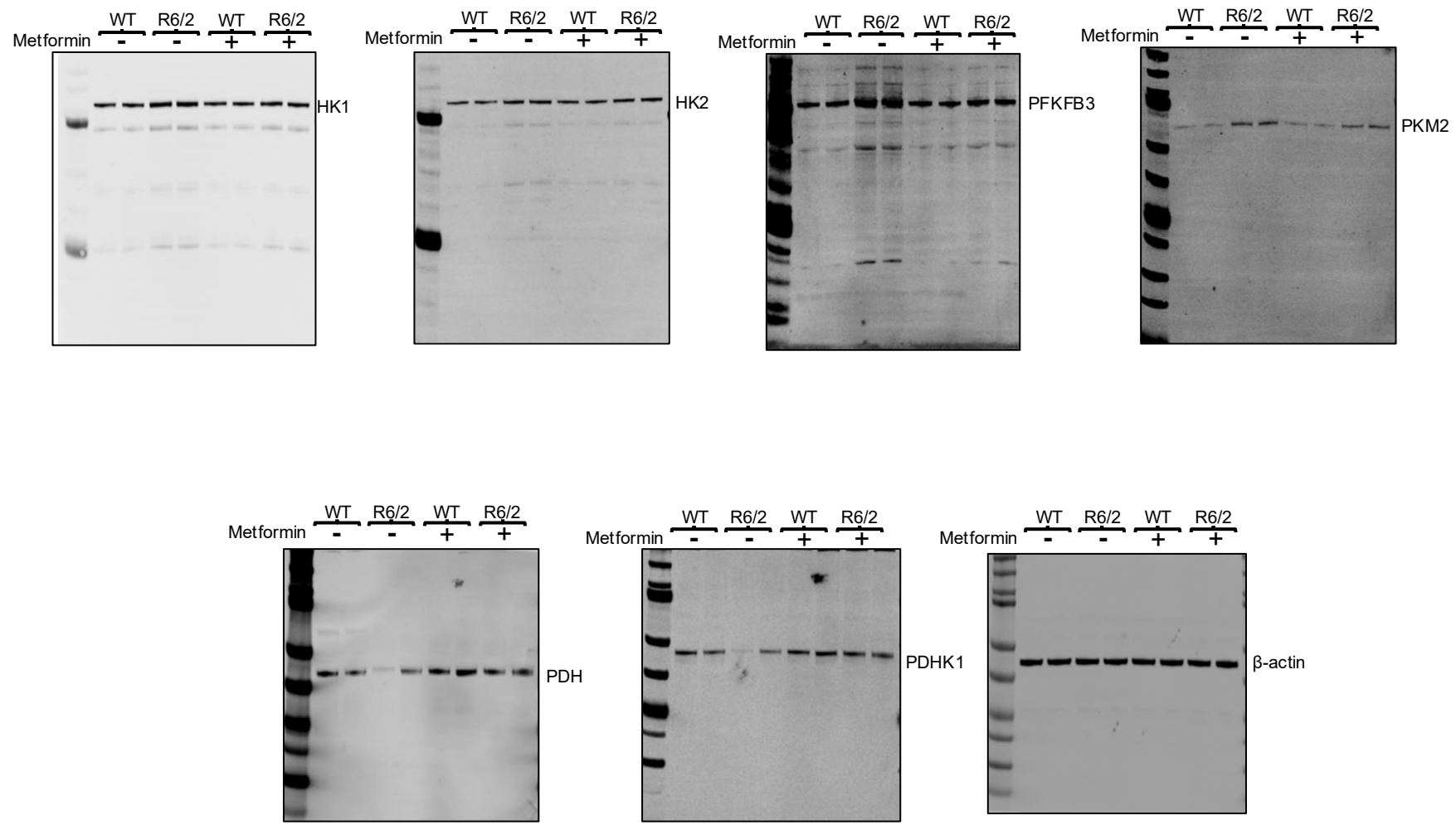

Figure 5A

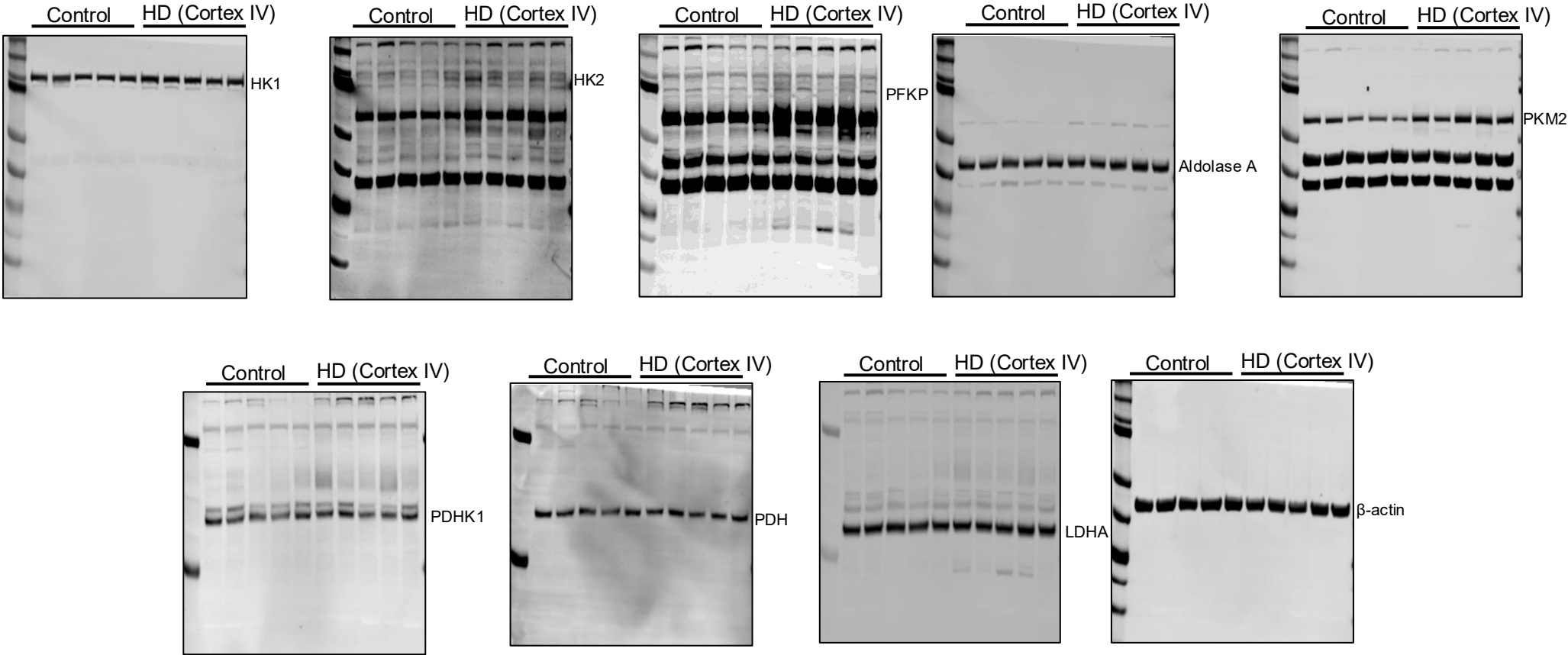

Figure 5C

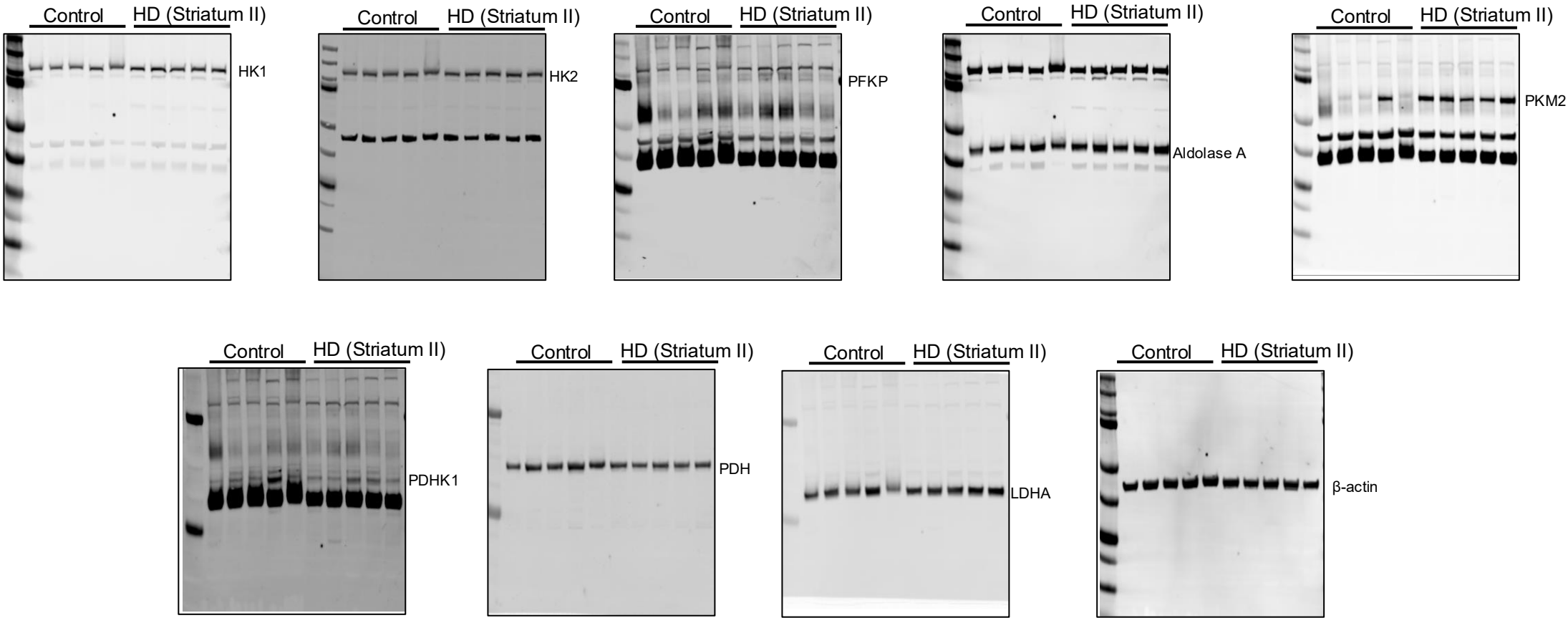

Figure 6E

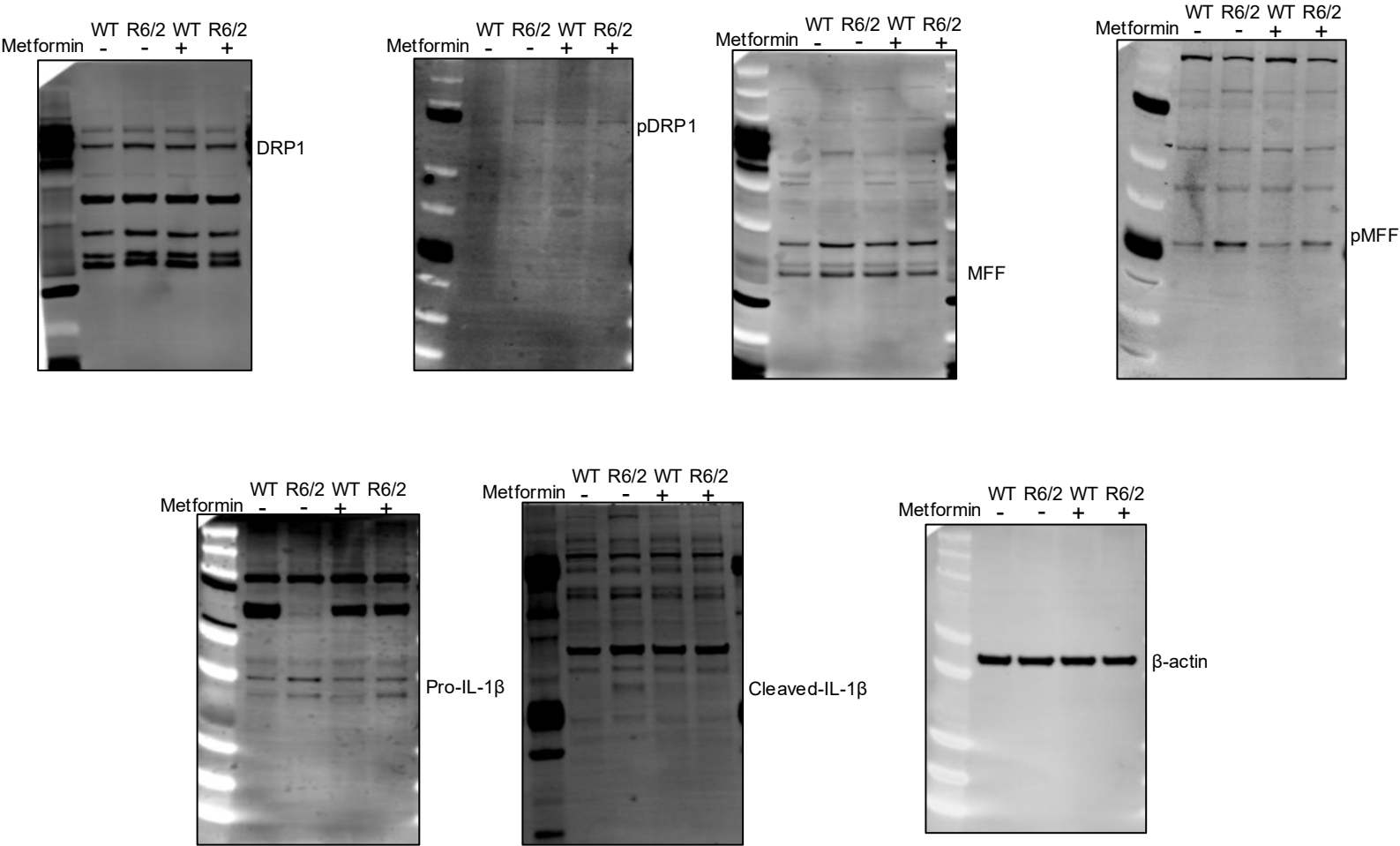

Supplement: Unedited blot and gel images [file jciinsight-11-201466-s150.pdf]
